# Cancer prognosis prediction using somatic point mutation and copy number variation data: a comparison of gene-level and pathway-based models

**DOI:** 10.1186/s12859-020-03791-0

**Published:** 2020-10-20

**Authors:** Xingyu Zheng, Christopher I. Amos, H. Robert Frost

**Affiliations:** 1 Department of Biomedical Data Science, Geisel School of Medicine, Dartmouth College, Hanover, NH 03755 USA; 2 Department of Medicine, Institute for Clinical and Translational Research, Baylor College of Medicine, 1 Baylor Plaza, Houston, TX 77030 USA

## Abstract

**Background:**

Genomic profiling of solid human tumors by projects such as The Cancer Genome Atlas (TCGA) has provided important information regarding the somatic alterations that drive cancer progression and patient survival. Although researchers have successfully leveraged TCGA data to build prognostic models, most efforts have focused on specific cancer types and a targeted set of gene-level predictors. Less is known about the prognostic ability of pathway-level variables in a pan-cancer setting. To address these limitations, we systematically evaluated and compared the prognostic ability of somatic point mutation (SPM) and copy number variation (CNV) data, gene-level and pathway-level models for a diverse set of TCGA cancer types and predictive modeling approaches.

**Results:**

We evaluated gene-level and pathway-level penalized Cox proportional hazards models using SPM and CNV data for 29 different TCGA cohorts. We measured predictive accuracy as the concordance index for predicting survival outcomes. Our comprehensive analysis suggests that the use of pathway-level predictors did not offer superior predictive power relative to gene-level models for all cancer types but had the advantages of robustness and parsimony. We identified a set of cohorts for which somatic alterations could not predict prognosis, and a unique cohort LGG, for which SPM data was more predictive than CNV data and the predictive accuracy is good for all model types. We found that the pathway-level predictors provide superior interpretative value and that there is often a serious collinearity issue for the gene-level models while pathway-level models avoided this issue.

**Conclusion:**

Our comprehensive analysis suggests that when using somatic alterations data for cancer prognosis prediction, pathway-level models are more interpretable, stable and parsimonious compared to gene-level models. Pathway-level models also avoid the issue of collinearity, which can be serious for gene-level somatic alterations. The prognostic power of somatic alterations is highly variable across different cancer types and we have identified a set of cohorts for which somatic alterations could not predict prognosis. In general, CNV data predicts prognosis better than SPM data with the exception of the LGG cohort.

## Background

Advances in high-throughput technologies have helped to identify and characterize the genomic landscape of human cancers. Large collaborative projects, such as The Cancer Genome Atlas (TCGA), have characterized gene expression, mutation, copy number, miRNA, and methylation features from over 20,000 primary cancers and adjacent normal samples spanning 33 cancer types [1]. Based on the analysis of these genomic features and clinical outcomes, many prognostic biomarkers have been proposed. Tumors that arise from the same tissue can behave heterogeneously and patients with the same cancer type can have variable clinical and genomic features. Therefore, patients can exhibit different prognoses. Cancer prognosis prediction can improve the stratification of patient risk, better personalize treatment and decrease unnecessary over-treatment [2]. Previous TCGA-wide genome-wide studies have often focused on gene expression features to identify cancer prognostic biomarkers [3–5]. Compared to analyses of cancer gene expression data, there have been fewer systematic reports on the association between somatic alterations and clinical outcomes such as patient survival [6]. Somatic alterations can be classified into two types: somatic point mutations (SPM), which include single nucleotide variants and indels which only affect one or a few genetic code letters, and somatic copy number variations (CNV), which involve larger contiguous portions of the genome either being lost (deletions) or duplicated (amplifications) [7]. A few studies have identified mutation features for specific cancer types, such as lung adenocarcinoma [8], acute myeloid leukemia [9], breast cancer [10] and colorectal cancer [11]. Most of these studies are conducted on a single cancer type with a single type of somatic alterations data. Additionally, most somatic alterations, even when aggregated at a gene-level, are too rare to support meaningful association studies. One alternative method is to summarize mutation information by certain features before conducting association studies [6]. One widely used feature for SPM is tumor mutation burden (TMB, the total number of SPMs). For CNV a similar measure is copy number alteration burden which indicates the degree to which a tumor’s genome is altered as a percentage of genome length [7]. But both of these measures are sample-wise measurements that give an overall score to each sample, therefore, both of them discard specific gene information. To more fully characterize alterations that jointly affect prognosis we propose using gene set enrichment methods to aggregate the information to the pathway-level so that a score is given for each pathway and each sample.

Gene set enrichment (GSE) analysis is a popular approach for condensing information from gene expression profiles into signature summaries. GSE methods evaluate statistics that are computed for biologically meaningful groups of genes [12], e.g., the sets defined in collections such as the Molecular Signatures Database (MSigDB) [13]. Most GSE methods, e.g., GSEA [13] and CAMERA (Correlation Adjusted MEan RAnk gene set test) [14], are supervised and population-level techniques, i.e., they evaluate the association between gene set statistics computed for an entire data set and some clinical outcome, e.g., case/control status. GSE methods also exist that can perform a so-called single sample analysis, i.e., they compute gene set statistics for each sample to transform a sample-by-gene matrix into a sample-by-pathway matrix. A number of single sample GSE methods have been developed for gene expression data, including single sample GSEA (ss GSEA) [15], Gene Set Variation Analysis (GSVA) [16] and Pathway Level Analysis of Gene Expression (PLAGE) [17].

To gain a global understanding of the prognostic power of somatic alterations, we systematically analyzed SPM and CNV data of 29 TCGA cancer types. We evaluated gene-level and pathway-level Cox proportional hazards models for just SPM data, just CNV data and the combination SPM and CNV data. Given the sparsity of somatic alterations data, it was our hypothesis that pathway-level models would have greater prognostic accuracy and be more interpretable, stable and parsimonious. We also evaluated a range of approaches for aggregating SPM data at the pathway level and different approaches for filtering genes prior to model estimation. Although the use of pathway-level predictors did not offer superior predictive power relative to gene-level models for all cancer types, we found that model robustness and parsimony are consistently better for pathway-level models. Our comprehensive analysis suggests that the prognostic power of somatic alterations is highly variable across different cancer types with low grade glioma offering the highest predictive accuracy. Based on the outcome of this comprehensive evaluation, we provide general recommendations for the use of gene-level versus pathway-level predictors and the use of SPM versus CNV data for cancer prognosis prediction.

## Methods

### Data sources

We downloaded the gene-level SPM, CNV data and clinical data from the UCSC Xena datahub [18] for 37 cohorts profiled by The Cancer Genome Atlas (TCGA) [1]. Among the 37 cohorts, 5 cohorts were removed because of an insufficient number of samples which are Bile Duct Cancer cohort (CHOL), Large B-cell Lymphoma cohort (DLBC), Formalin Fixed Paraffin-Embedded Pilot Phase II cohort (FPPP), Skin Cutaneous Melanoma (SKCM) and Uterine Carcinosarcoma cohort (UCS). Three combinations of the subtype cohorts were also excluded which are colon and rectum adenocarcinoma (COADREAD), brain lower grade glioma and glioblastoma multiforme (GBMLGG) and lung cancer (LUNG). After these removals, there were 29 cohorts available for analysis. The characteristics including sample size and death rate of the 29 cohorts are provided in the Additional file 1: Table X1.

For the SPM data, we downloaded the dataset from the UCSC Xena datahub, which has processed the variants to gene-level binary values. The wrangling steps conducted by UCSC Xena for this dataset include: (1) download mc3.v0.2.8.PUBLIC.maf.gz, (2) only keep mutations with filter = PASS, (3) convert to binary gene-level non-silent mutation calls, (4) extract cohort sample data. Specifically, mutation calls were produced by the Multi-center Mutation Calling in Multiple Cancers (MC3) working group and were published in file *mc3.v0.2.8.PUBLIC.maf.gz* (https://api.gdc.cancer.gov/data/1c8cfe5f-e52d-41ba-94da-f15ea1337efc) [19]. The MC3 efforts provide consensus calls from 7 software packages and they also provide a “PASS” identifier to indicate whether the variant pass the filter criteria. The MC3 efforts took significant steps to remove potential germline calls and non-exonic variants. Filter flags include low normal depth coverage, non-exonic sites, sites outside of capture kit, sites marked by the Broad Panel of Normals, samples marked as being contaminated by ContEst, and variants that were only called by a single caller. If a mutation was not assigned any flag, it received a ‘PASS’ identifier [20]. Based on this identifier, only the variants with filter = PASS were kept and they were converted to gene-level binary values indicating whether there are non-silent mutations. For the CNV data, we downloaded the dataset from UCSC Xena datahub, which has been processed to gene-level estimates. Specifically, the copy number profile was measured experimentally using whole genome microarray at a TCGA genome characterization center. Subsequently, TCGA FIREHOSE pipeline applied GISTIC2 method [21] to produce segmented CNV data, which was then mapped to genes using UCSC Xena HUGO probeMap to produce gene-level estimates. During this process, filtering steps included: (1) probe sets that were previously indicated to be associated with frequent germline copy-number variation were removed, (2) only protein-coding genes were kept.

The HALLMARK pathway collection (50 pathways), REACTOME (1499 pathways), PID (196 pathways derived from Pathway Interaction Database), BIOCARTA (289 pathways) from C2 collection and BP from C5 collection (1350 pathways), were obtained from the Molecular Signatures Database (MSigDB) [13]. Detailed information about the used pathway collections is provided in Additional file 1: Table X2.

A list of all cancer census genes was downloaded from the COSMIC website (723 genes) (release v90, 5th September 2019) [22]. This list was used for further filtering genes to be used in the analyses.

### Prognostic models

**Fig. 1:**
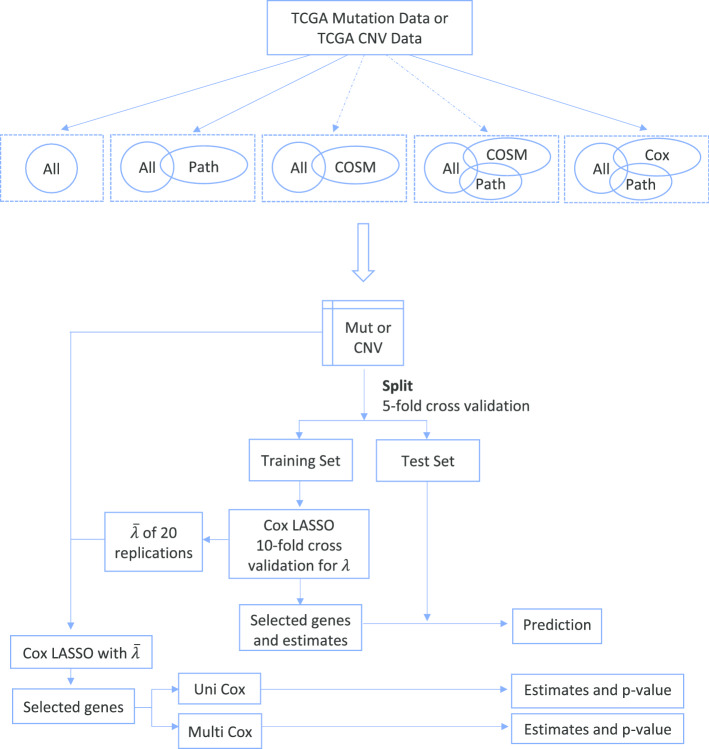
Workflow of gene-level models. In the 5 boxes are 5 intersected sets we achieved after filtering. “All” represents all the genes in the data, “Path” represents all the genes in the pathway collection, “COSM” represents all the genes in the COSMIC database, “Cox” represents all the genes which have significant *p* value in the univariable Cox model. The gene subsets represented by the 2 boxes after the dashed arrows were only applied to SPM data. 5-fold cross validation is conducted and for each training set, Cox Lasso is fitted. The estimated Cox model is applied to the test set to assess predictive performance. Unpenalized Cox models are also fitted, with the average value of lambda as the regularization parameter, to obtain non-shrunken coefficient estimates and *p* values

Figure 1 is the workflow of gene-level models. In addition to models that used all of the genes in the TCGA data, we also tried several criteria for filtering genes prior to model estimation: only include genes that are contained in the target pathway collection (we only used the MSigDB Hallmark collection in this case to avoid an overly complex comparison), only include genes in the COSMIC cancer gene census (this filtering was only performed for SPM data), only include genes meeting both the pathway collection and COSMIC criteria, and only include genes in the target pathway collection that also have a significant association with survival according to a univariate Cox model (*p* value $\le \hspace{0.17em}$0.05). To avoid a biased estimation of predictive performance, filtering based on the results from a univariate Cox model was performed on the training data, which comprised 80% of the samples. Filtering the gene-level predictors according to both the COSMIC cancer gene census and the results from univariable Cox models indirectly account for risk level and functional impact of the associated variants.

After variable filtering, we performed 20 iterations of nested cross-validation to evaluate the predictive performance of each model. Specifically, a fivefold cross validation randomly splits the data into training and test sets with 4/5 proportion of the samples for training and 1/5 proportion for testing. For each training set, tenfold cross validation is conducted within Cox Lasso to choose the tuning regularization parameter lambda value that gives minimum mean cross-validated error. With the amount of regularization controlled by the selected lambda, the estimated Cox model is applied to the test set to assess predictive performance as quantified by the concordance index (see the “Model evaluation metrics” section for details). Using the average of the 100 chosen lambda values (20 replications multiplied by 5 cross validation folds) as the regularization parameter, we fit a Cox Lasso model on the whole dataset and retained the variables with non-zero coefficients. With these variables, we estimated both an unpenalized multivariate Cox model and, for each retained variable, a univariate Cox model to obtain non-shrunken coefficient estimates and *p* values.

In our analysis, we adopted Cox regression models, for both multivariable and univariable analyses. Cox regression models are the most widely used method for survival analysis with censored data and several studies have shown that Cox regression models are at least as good as, or even better than, neural networks, SVMs, random survival forest and other machine learning methods when modeling censored survival with clinical variables [23–25]. But when considering the high dimensionality issue of omics data in which the number of covariates is larger than the sample size, Cox regression models encounter the over-fitting issue. Under this situation, combining Cox regression models with a Lasso penalty for variable selection is widely used to identify prognostic biomarkers and obtain more parsimonious models.

**Fig. 2:**
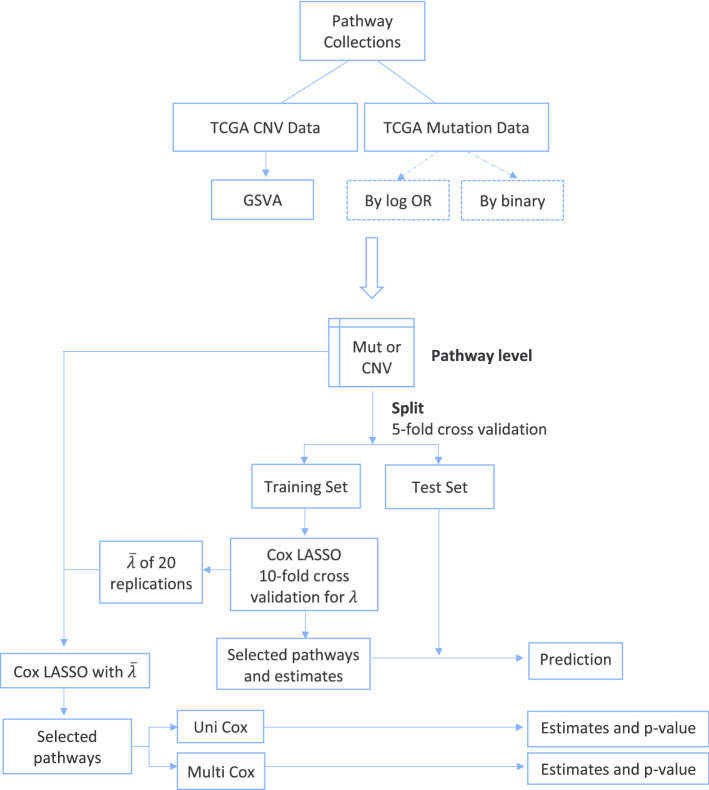
Workflow of pathway-level models. The abbreviation “Uni Cox” represents “Univariable Cox model”, “Multi Cox” represents “Multivariable Cox model”. 5-fold cross validation is conducted and for each training set, Cox Lasso is fitted. The estimated Cox model is applied to the test set to assess predictive performance. Unpenalized Cox models are also fitted, with the average value of lambda as the regularization parameter, to obtain non-shrunken coefficient estimates and *p* values

Figure 2 displays the workflow for evaluation of the pathway-level models. The gene-level variables are first transformed to pathway-level variables. In this study, we performed a single sample GSE analysis of TCGA somatic alterations data rather than a population-level analysis since we aim to use either gene-level or pathway-level somatic alterations statistics as predictor variables in Cox proportional hazards models. An important limitation of current GSE methods like GSVA is that they were designed for gene expression data and have not been evaluated on CNV and SPM data. In this study, we directly applied the current GSVA implementation to CNV data. GSVA conducts kernel density estimation of the cumulative distribution function and Kolmogorov–Smirnov (KS) like random walk to calculate sample-level statistics. We expect that the approach taken by GSVA will work similarly on both gene expression data and the continuous TCGA level 3 CNV data. A difference between SPM and CNV is that CNVs typically affect multiple genes in a contiguous region and are therefore more affected by the regional organization of genes. For SPM data, a straightforward method for computing pathway-level statistics is to count the number of mutated genes in each pathway [26]. In this study, we use two different methods for calculating pathway-level values from SPM data, which we refer to as the log-odds ratio method and the binary method. For the log-odds ratio approach, a 2-by-2 table is created for each pathway and sample by counting the number of genes in each of the four possible combinations (being in the specific pathway or not and being mutated or not). Using this 2-by-2 table, the odds ratio is computed to measure the association between being in the specific pathway and being mutated. The log-odds ratios are used as predictor values for pathway-level models. For the binary approach, for each pathway and sample, if there are mutated genes in this pathway, the value is 1, otherwise it is 0. Once the pathway-level data matrix has been generated, evaluation of prognostic ability follows the same steps outlined above for the gene-level models.

In addition to the SPM only and CNV only models, we also investigated integrated models that used both SPM data and CNV data. For these evaluations, we used both SPM and CNV-based predictors defined at either the gene-level or pathway-level and assessed predictive performance using the same approach. For the gene-level integrated models, gene-level SPM and CNV data were combined and then analyzed using the workflow shown in Fig. 1. For the pathway-level integrated models, pathway-level SPM and CNV data were combined and then analyzed using the workflow shown in Fig. 2.

### Model evaluation metrics

The concordance index (CI), Fleiss kappa statistic and average number of predictors were implemented as evaluation metrics in this study, which are same with our previous study in [27]. We used the average concordance index ranging between 0 and 1, to quantify the predictive power of each model. The concordance index, or c-index, is one of the most widely utilized performance measures for survival models which can be interpreted as the concordance between the prediction and the survival outcomes. Specifically, a CI of 1 indicates perfect prediction accuracy and a CI of 0.5 represents random prediction [28]. The Fleiss kappa statistic [29] is exploited to evaluate the repeatability and stability of models. The Fleiss kappa statistic is frequently utilized to test interrater reliability with 1 indicating perfect agreement and 0 indicating no agreement. Measurement of the extent to which raters assign the same score to the same variable is called the interrater reliability [30]. In our case, each trained model is designed to be a rater to assign the affiliation of each variable (gene or pathway). We conducted 20 replications of fivefold cross validation. As such, we had 100 trained models, or 100 raters in total, among which the agreement was measured by the Fleiss kappa. Finally, we used the average number of predictors in the 100 trained models to measure model parsimony.

### Null models

To ensure the predictive signals are not generated randomly and that the prediction is not inflated in our analyses, we checked the results of null models, in which all the steps are the same except that the survival outcomes are shuffled among individuals to break any association between the variables and the outcomes, while maintain the correlations among variables. The result of these null models in Additional file 1: Figure S1 and show that for all the models and cohorts, the concordance is around the expected null value of 0.5, which demonstrates that the signals in our true models are valid.

### Simulation study for Lasso

To show how Lasso works when there are duplicated variables (perfect collinearity) in the data, we designed a simple simulation study as described in Additional file 1.

## Results

### Across cancer types

To have a better understanding about the comparison of different workflows across different cancer types, we plotted heatmaps of the concordance index (predictive power), Fleiss kappa statistics (robustness) and average model size (parsimony).

**Fig. 3:**
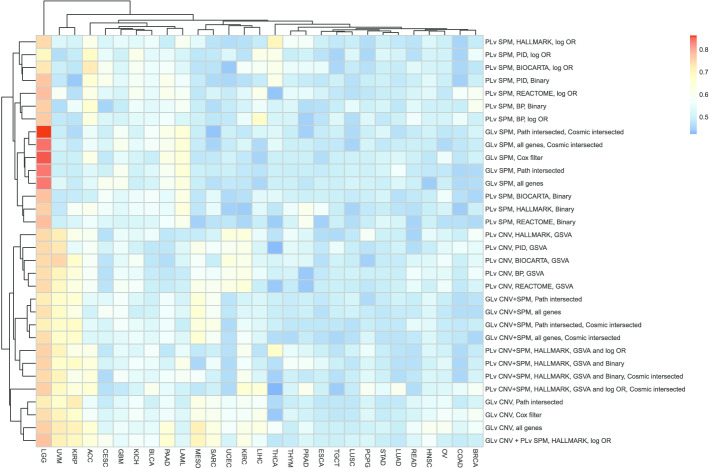
Heatmap of the concordance index for different models and cancer types. “PLv” represents “Pathway-level” and “GLv” represents “Gene-level”. HALLMARK, PID, BIOCARTA, REACTOME and BP are the used five pathway collections as introduced in “Methods” section. Corresponding to each model introduced in “Methods” section: “log OR” and “Binary” represent the two enrichment methods for SPM data; “all genes”, “Path intersected”, “Cosmic intersected”, “Cox filter” represents the filters on the genes. The maximum value among all is 0.86 and the minimum is 0.42

Figure 3 plots the concordance index between predicted and observed outcomes across different models and cancer types. The LGG cohort performed remarkably well for all models, especially for the gene-level SPM models. While for cohorts such as UVM and KIRP, the SPM-only models have close to null predictive power using either gene-level or pathway-level predictors. It is interesting to note that the SPM models are clustered separately from the CNV models and SPM/CNV combination models, which shows that SPM and CNV data provide distinct information regarding cancer prognosis. The comparison between the log-odds ratios approach and the binary approach shows that the log-odds ratios approach is slightly better than the binary approach although it did not significantly improve the predictive performance. The comparison across different pathway collections and different intersected gene filtering shows that adopting different pathway collections or different filtering methods did not improve the predictive performance.

Figure 3 also shows that for the cohorts clustered on the right half (from THCA to BRCA), none of the models works well. It indicates that the prediction for cancer prognosis is cancer type dependent. A similar conclusion has been reported in [31]. This may be due to the fact that somatic alterations data may not predict patient prognosis for these cancer types. For example, for lung cancer (LUSC and LUAD), clinical characteristics such as smoking status or stage may be a more important factor in determining patient survival. The poor predictive performance of these models may also be due to poor quality survival data, such as for breast cancer (BRCA), using overall survival endpoint was cautioned against by [32] due insufficient follow-up, or a low death rate, such as for PCPG, TGCT, PRAD, THYM, THCA (death rates are 0.04, 0.03, 0.02, 0.07, 0.03 respectively as shown in Additional file 1: Table X1).

**Fig. 4:**
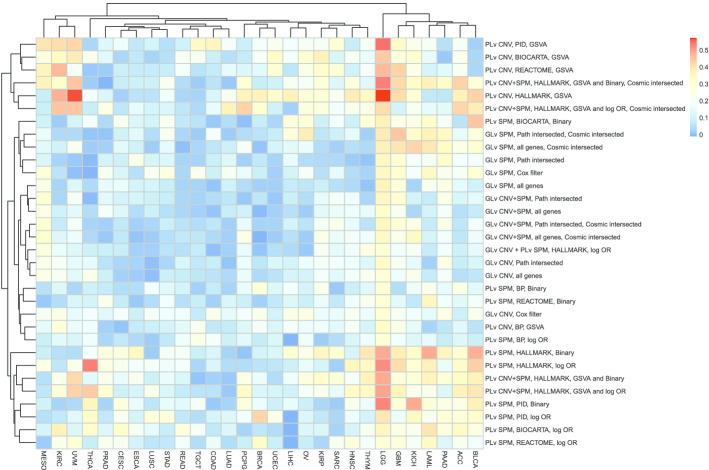
Heatmap of the Fleiss kappa statistic for different models and cancer types. “PLv” represents “Pathway-level” and “GLv” represents “Gene-level”. HALLMARK, PID, BIOCARTA, REACTOME and BP are the used five pathway collections as introduced in "Methods" section. Corresponding to each model introduced in "Methods" section: “log OR” and “Binary” represent the two enrichment methods for SPM data; “all genes”, “Path intersected”, “Cosmic intersected”, “Cox filter” represents the filters on the genes. The maximum value among all is 0.57 and the minimum is − 0.01

Figure 4 plots the Fleiss kappa statistic, which measures the agreement of replicates about the selection of predictors, for different models and cancer types. Based on the clustering results, it is clear that the pathway level models have higher Fleiss kappa values than the gene-level models although there are several exceptions. Both C2 REACTOME and C5 BP are large pathway collections (1499 and 1350 pathways respectively), while there are only 723 genes in the COSMIC cancer gene census (711 of them are in the SPM data and 326 are also in the Hallmark pathway collection). Thus, it is reasonable that the pathway-level models with the REACTOME and C5 BP pathways are less stable than gene-level models with only COSMIC genes for some cohorts. In these cases, more variables are entering the Lasso model selection with the pathway-level analysis than for the restricted gene-level analysis.

Figure 4 also shows that models fit on the LGG cohort, especially the pathway-level models, are the most robust for all evaluated cancer types and modeling approaches. For some cohorts (the middle left area, from THCA to OV), the Fleiss kappa statistic is close to 0, i.e., equivalent to random guessing. These cohorts overlap with the cohorts in the right half in Fig. 3 (12 out of 15 from Fig. 4 and 12 out of 14 from Fig. 3 are the same), which implies that there is an association between the prediction concordance index and model stability. For these cohorts, the models tend to randomly choose some predictors with poor predictive performance, which may be due to insufficient somatic alterations information or poor survival data quality or insufficient events for reliable inference.

**Fig. 5:**
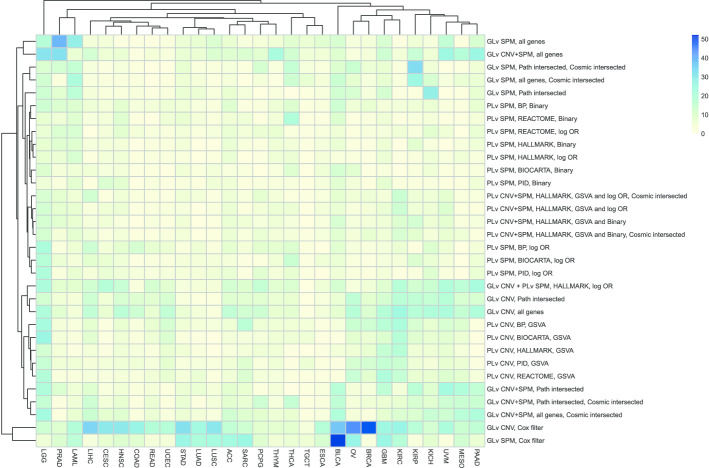
Heatmap of average model sizes for different models and cancer types. “PLv” represents “Pathway-level” and “GLv” represents “Gene-level”. HALLMARK, PID, BIOCARTA, REACTOME and BP are the used five pathway collections as introduced in "Methods" section. Corresponding to each model introduced in "Methods" section: “log OR” and “Binary” represent the two enrichment methods for SPM data; “all genes”, “Path intersected”, “Cosmic intersected”, “Cox filter” represents the filters on the genes. The maximum value among all is 52.19 and the minimum is 0.25

Figure 5 plots the average number of predictors for different models and cancer types. The pathway-level models cluster separately from other models and are more parsimonious than the gene-level models.

### Representative cohort

**Fig. 6:**
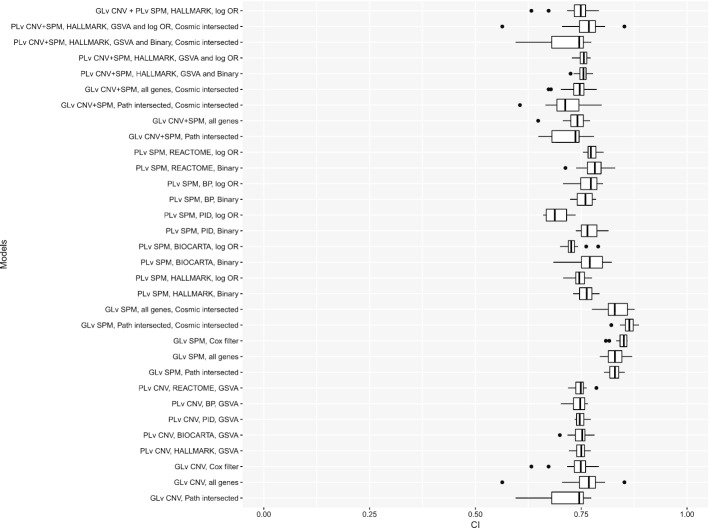
Distribution of concordance index values for all models evaluated on the LGG cohort. “PLv” represents “Pathway-level” and “GLv” represents “Gene-level”. HALLMARK, PID, BIOCARTA, REACTOME and BP are the used five pathway collections as introduced in "Methods" section. Corresponding to each model introduced in "Methods" section: “log OR” and “Binary” represent the two enrichment methods for SPM data; “all genes”, “Path intersected”, “Cosmic intersected”, “Cox filter” represents the filters on the genes

Based on predictive performance, model stability and model parsimony, LGG is unique among the 29 analyzed TCGA cohorts. For the Lower Grade Glioma (LGG) cohort, the death rate is 0.253 and the sample size is 508. Figure 6 illustrates the distribution of concordance index values for all models estimated on the LGG cohort; equivalent figures for the other cohorts are provided in Additional file 1: Figure S2-S30. As seen in Fig. 6, predictive power is good for all model types on the LGG cohort (the median is as high as 0.75) with the best performance obtained by the gene-level SPM models. To better understand the biological basis for the strong predictive performance of the LGG models, we investigated the predictors used in the representative gene-level and pathway-level models estimated using both SPM and CNV data.

Table 1 lists the predictors used in the SPM gene-level (all genes) model which we fit on LGG SPM data using all available genes. For this model, the concordance index was 0.84 (± 0.02). As described in the “Methods” section, coefficient estimates and *p* values in Table 1 are from unpenalized multivariable and univariable Cox models.

**Table 1:** Predicotors of LGG SPM gene-level (all genes) model and fitting results of Cox models

	Multivariable Cox model			Univariable Cox model		
	Hazard ratios	se(coef)	*p* value	Hazard ratios	se(coef)	*p* value
PDGFRA	29.73	0.49	5.47E−12	24.49	0.48	2.66E−11
IDH1	0.28	0.22	5.68E−09	0.25	0.18	5.93E−14
EGFR	4.17	0.29	6.15E−07	5.05	0.25	1.23E−10
KALRN	0.22	1.04	1.46E−01	0.15	1.02	6.76E−02
TNR	0.00	2402.16	9.90E−01	2.91E−08	2560.82	9.95E−01
GALNT12	0.00	1249.74	9.90E−01	0.20	1.03	1.23E−01
SERINC3	0.00	2402.16	9.90E−01	2.91E−08	2560.82	9.95E−01
TMX4	0.00	2235.75	9.94E−01	9.57E−08	1683.51	9.92E−01
CDK19	0.00	7374.16	9.98E−01	3.29E−08	2381.56	9.94E−01
TMEM82	1.61	7732.63	1.00E+00	3.21E−08	2611.31	9.95E−01
TWISTNB	1	0	NA	2.91E−08	2560.82	9.95E−01
UTP6	1	0	NA	0.20	1.03	1.23E−01
ACTL8	1	0	NA	2.91E−08	2560.82	9.95E−01
TLR3	1	0	NA	2.91E−08	2560.82	9.95E−01
PPP1R16B	1	0	NA	3.21E−08	2611.31	9.95E−01
FLAD1	1	0	NA	2.91E−08	2560.82	9.95E−01

The three genes associated with significant predictors all have an established association with glioma. The EGFR gene mutant is highly oncogenic [33]. Amplification and overexpression of EGFR are a particularly striking feature of glioblastoma (GBM), observed in approximately 40% of tumors. Although PDGFRA amplification is less common in gliomas than EGFR amplification, PDGFRA gene amplification is found in 11% of GBMs, making it the second most frequent RTK gene amplified in this family of tumors. Two activating PDGFRA gene rearrangements in gliomas have been identified and suggest the possibility that these PDGFRA mutants behave as oncogenes [34]. Besides glioblastoma, amplified PDGFRA and EGFR may also occur in lower-grade gliomas and in their recurrent tumors [35]. Mutations in IDH1 are often the first hit in the development of diffuse gliomas, suggesting IDH1 mutations as key events in the formation of these brain tumors [36]. These results support the predictive and interpretative power of the gene-level SPM model for the LGG cohort.

There are several important limitations of the gene-level SPM model. First, for 6 of the genes selected by LASSO, the unpenalized multivariate Cox model gave null estimates and *p* values. This was caused by the collinearity of these genes with other genes retained in the model. Due to the extreme sparsity of the SPM data, such as for the LGG cohort where 99.9% of the entries in the SPM data matrix are 0, it is possible to find groups of genes that are mutated in the same small set of patients, e.g., two genes both mutated in just one patient. In this situation, the unpenalized Cox proportional hazards model, as implemented by the R coxph() function in the survival package [37], will report *p* values as NA and estimated coefficients as 0. We were surprised that the LASSO penalized model in this scenario retained multiple highly collinear predictors given the conventional wisdom that LASSO will tend to retain just one from a group of correlated predictors [38]. A simple simulation study was designed to show that LASSO was not guaranteed to discard duplicated variables no matter which lambda chosen and the results are provided in Additional file 1: Figure S31. One approach to decrease the collinearity issue in this situation is adding the constraint to require a certain number of mutations in a gene for it to enter the model. For example, filtering the genes with more than 2 mutations before fitting the model would largely decrease the number of genes with high collinearity. The second key limitation of gene-level SPM models is that some genes retained by LASSO (TNR, GALNT12, SERINC3, TMX4, CDK19, TMEM82 in Table 1) have very large coefficient standard errors in the unpenalized model. For the LGG cohort, this is because these genes are only mutated in a small number of patients, most of whom have a censored survival status, which results in highly variable coefficient estimates. Among the 6 genes from Table 1, 5 genes are mutated in only 2 patients and another one was mutated in just 3 patients. Among those patients, only one patient had a death event.

Table 2 lists the predictors retained by LASSO for the pathway-level LGG SPM model where predictor values were generated using log-odds ratios for the pathways in the MSigDB C2 REACTOME collection. For this model, the concordance index was 0.78 (± 0.01). The coefficient estimates and *p* values from unpenalized multivariable and univariable Cox models are also included in Table 2.

**Table 2:** Predictors of LGG SPM pathway-level (C2 REACTOME) model and fitting results of Cox models

	Multivariable Cox model			Univariable Cox model		
	Hazard ratios	se(coef)	*p* value	Hazard ratios	se(coef)	*p* value
TCA_CYCLE_AND_RESPIRATORY_ELECTRON_TRANSPORT	0.24	0.16	5.82E−19	0.54	0.08	4.03E−15
SIGNALING_BY_CONSTITUTIVELY_ACTIVE_EGFR	1.79	0.17	7.24E−04	1.03	0.11	7.81E−01
G_ALPHA_Q_SIGNALLING_EVENTS	0.58	0.17	1.00E−03	0.62	0.14	5.09 E−04
PROTEOLYTIC_CLEAVAGE_OF_SNARE_COMPLEX_PROTEINS	2.64	0.31	1.58 E−03	0.87	0.08	7.50 E−02
DNA_REPLICATION	0.56	0.28	3.74 E−02	0.47	0.15	4.92 E−07
OLFCTORY_SIGNALING_PATHWAY	1.31	0.13	4.78E−02	0.94	0.12	6.05E−01
CELL_CYCLE_MITOTIC	0.95	0.21	8.14E−01	0.52	0.15	2.58E−05

Although the concordance index for the pathway-level model is lower than the value for the gene-level model of Table 1 (0.78 vs 0.84), aggregation of SPM data based on pathways avoids the sparsity and collinearity issues encountered by the gene-level model. Most of the selected REACTOME pathways are consistent with the genes selected by the gene-level models. The TCA (tricarboxylic acid) cycle is associated with IDH1/2 mutation status due to the fact that mutation of IDH alters the intermediate metabolite α-ketoglutarate (αKG) in the TCA cycle [39]. REACTOME_SIGNALING_BY_CONSTITUTIVELY_ACTIVE_EGFR has an obvious association with EGFR. G alpha (q) is one type of G protein, which plays an important role in the function of G protein-coupled receptors (GPCR) [40]. GPCRs constitute a large family of membrane receptors affecting oncogenic pathways via canonical and non-canonical signaling [41] and are the targets of more than 30% of cancer drugs [42]. DNA_REPLICATION and the CELL_CYCLE_MITOTIC are also highly related with tumorigenesis. CELL_CYCLE_MITOTIC was not significant in multivariable Cox model while it was significant in univariable Cox model. This may be due to its correlation with DNA_REPLICATION in multivariable Cox model. In the REACTOME pathway collection, CELL_CYCLE_MITOTIC includes all the genes in the DNA_REPLICATION.

The pathway-level models not only provide better interpretative power, they are also more stable and have more reasonable coefficient estimates because they suffer less from collinearity. Some predictors from the gene-level models can have abnormally large coefficient estimates, which are driven by collinearities induced by insufficient numbers of mutations in some genes. These coefficients may not be reliable, as indicated by their very large standard errors.

In addition to the MSigDB C2 REACTOME collection (1499 pathways), we also investigated the MSigDB Hallmark (50 pathways) and C5 BP (1350 pathways) collections. The Hallmark pathways retained in the LGG SPM model include protein secretion, bile acid metabolism and xenobiotic metabolism, as shown in Additional file 1: Table X3 and X4. The Hallmark protein secretion pathway includes EGFR, which has a known association with glioma as described above. The Hallmark bile acid metabolism pathway includes IDH1 and IDH2, which also have known glioma associations. Bile acid biosynthesis produces metabolites known to induce apoptosis and inhibit cancer cell proliferation [43]. The Hallmark xenobiotic metabolism pathway includes IDH1 and involves Cytochrome P450 enzymes. The recent research shows that Cytochrome P450 enzymes (P450s) have become important targets in cancer analysis as their role in xenobiotic metabolism. These enzymes can function in either inactivating carcinogens or generating reactive moieties leading to carcinogenesis [44].

Table 3 includes the list of predictors used in the LGG CNV gene-level (all genes) model, including the chromosomal locations of genes. For this model, the concordance index was 0.73 (± 0.09). Similar to Tables 1 and 2, Table 3 includes coefficient estimates and *p* values from unpenalized multivariable and univariable Cox models.

**Table 3:** Predictors of LGG CNV gene-level (all genes) model and fitting results of Cox models

	Multivariable Cox model			Univariable Cox model		
	Hazard ratios	se(coef)	*p* value	Hazard ratios	se(coef)	*p* value
METTL1 (12q14.1)	1.37	0.10	1.55E−03	1.75	0.08	1.49E−12
JPH4 (14q11.2)	0.36	0.37	5.84E−03	0.11	0.39	1.45E−08
SLC16A9 (10q21.2)	0.29	0.58	3.18E−02	0.04	0.27	1.01E−30
NRG3 (10q23.1)	0.07	1.95	1.62E−01	0.05	0.26	4.47E−31
MTAP (9p21.3)	0.30	1.03	2.35E−01	0.13	0.21	1.82E−22
CCSER2 (10q23.1)	12.94	2.68	3.39E−01	0.04	0.27	3.83E−31
ZC3H7B (22q13.2)	0.66	0.76	5.87E−01	0.17	0.30	3.56E−09
LINC00864 (10q23.2)	0.58	1.64	7.40E−01	0.05	0.27	2.40E−29
TNRC6B (22q13.1)	0.74	1.30	8.18E−01	0.11	0.31	3.04E−12
C9orf53 (9p21.3)	0.83	1.02	8.51E−01	0.13	0.21	1.53E−22
APOBEC3F (22q13.1)	1.19	1.10	8.71E−01	0.10	0.32	5.10E−13
KLLN (10q23)	0.96	0.96	9.64E−01	0.06	0.25	1.95E−29
LINC00948 (10q21.2)	NA	0	NA	0.04	0.27	1.01E−30
CCDC6 (10q21.2)	NA	0	NA	0.04	0.27	1.01E−30
C10orf40 (10q21.2)	NA	0	NA	0.04	0.27	1.01E−30
APOBEC3G (22q13.1)	NA	0	NA	0.10	0.32	5.10E−13

In this case, the genes with NA estimates in the multivariable Cox model are exactly collinear with other genes in the model. It is interesting to note that all of the genes selected by LASSO have significant *p* values in univariable Cox models, but some have insignificant *p* values in the multivariable Cox model. This may be due to the correlation with other variables in multivariable Cox model.

Table 4 includes the list of predictors used in the LGG CNV pathway-level model, with predictor values generated using the GSVA method for pathways in the MSigDB Hallmark collection. For this model, the concordance index was 0.75 (± 0.01). Similar with Table 3, some variables were not significant in multivariable Cox model while they were significant in univariable Cox model. This may be due to the correlation with other variables in multivariable Cox model.

**Table 4:** Predictors of LGG CNV pathway-level (Hallmark) model and fitting results of Cox models

	Multivariable Cox model			Univariable Cox model		
	Hazard ratios	se(coef)	*p* value	Hazard Ratios	se(coef)	*p* value
HYPOXIA	2.59E−03	1.73	5.79E−04	0.04	1.15	5.24E−03
MYC_TARGETS_V1	507.05	2.16	3.85E−03	858.29	1.27	9.38E−08
CHOLESTEROL_HOMEOSTASIS	13.96	1.08	1.49E−02	3.58	0.73	7.94E−02
PI3K_AKT_MTOR_SIGNALING	25.10	1.37	1.86E−02	2043.83	0.97	4.75E−15
OXIDATIVE_PHOSPHORYLATION	0.02	1.77	2.66E−02	0.03	1.34	7.16E−03
TGF_BETA_SIGNALING	6.40	0.96	5.33E−02	42.35	0.61	7.89E−10
EPITHELIAL_MESENCHYMAL_TRANSITION	11.00	1.79	1.79E−01	29.69	0.92	2.15E−04
IL2_STAT5_SIGNALING	5.36	1.52	2.69E−01	3.74	1.21	2.73E−01
KRAS_SIGNALING_UP	4.08	1.55	3.65E−01	151.12	0.86	5.18E−09
DNA_REPAIR	3.07	1.36	4.10E−01	15.60	1.08	1.12E−02
MYC_TARGETS_V2	0.42	1.19	4.70E−01	0.38	0.77	2.14E−01
ESTROGEN_RESPONSE_LATE	0.34	1.81	5.55E−01	0.01	1.18	1.35E−04
HEME_METABOLISM	0.39	1.80	6.01E−01	0.01	1.47	2.40E−03
HEDGEHOG_SIGNALING	0.75	0.70	6.82E−01	0.08	0.54	2.96E−06
ESTROGEN_RESPONSE_EARLY	1.31	1.96	8.90E−01	1.94E−03	0.90	3.78E−12
NOTCH_SIGNALING	1.04	0.75	9.54E−01	9.07	0.58	1.34E−04

## Discussion

The main aim of this study was to evaluate SPM/CNV data for prognosis prediction in a pan-cancer setting on both the gene and pathway levels. It was not aimed at finding the best modeling approach (i.e., comparison of penalized Cox models with other statistical approaches for survival prediction) nor focused on just pathway-level models. Instead, we aimed to systematically evaluate and compare SPM data and CNV data, gene-level models and pathway-level models in a pan-cancer setting. Based on our results on three dimensions of cancer prognosis prediction (predictive power, stability and parsimony), the low-grade glioma (LGG) cohort had markedly superior performance relative to the other evaluated TCGA cohorts. The median CI across all models is 0.75 with CI values as high as 0.85 for some gene-level models. Additionally, the models for the LGG cohort have high stability across replicates and good parsimony, meaning they use just a few somatic alterations features to predict prognosis well and the choice of predictors is robust across replications. This finding indicates that using genomic features, even just somatic alterations features, can be practical for predicting a LGG patient’s survival. It also narrows down a potential interesting list of genes or pathways for down-stream experiment to investigate the underlying mechanisms related to survival. Eventually these studies could lead to targeted therapies. Findings of our methods are validated by finding that for LGG, IDH1 and IDH2 mutations are selected, given the well-known prognostic value of mutations in these genes for predicting glioma survival [45].

During the investigation of specific predictors used in the models, we found that collinearity (often perfect collinearity) is a serious issue for the gene-level SPM models while pathway-level models largely reduce this issue. The SPM data is extremely sparse and some genes are only mutated in one or two patients, thus some patients can have exactly nearly or exactly the same mutation profiles for selected genes. For the CNV data, different genes located in the same region may be called together and share the same values. Therefore, the collinearity issue is a serious and common problem for both SPM and CNV data. Since the pathway-level variables are the statistics computed from a group of gene-level statistics, they suffer less from collinearity. Pathway level analysis avoids perfect collinearity because it is impossible that all the genes in a pathway have the same values. As we saw by the large coefficients, collinearity can make the parameter estimates unstable, so that standard errors of estimates are inflated, which can lead to biased estimation [46]. These large inflations may make inference statistics biased and certainly less likely to be reproducible, since they reflect effects from a limited number of patients.

Because of the collinearity issue in somatic alterations data, we noticed that Lasso was not guaranteed to discard duplicated variables or variables with high correlation. We also showed this in a simple simulation study, which is described in Additional file 1. This finding surprised us since it is widely accepted that when variables are highly correlated, Lasso will randomly retain one of them. However, our results show that this characteristic is not guaranteed. The issue of Lasso inconsistency has been discussed in some studies [47, 48] and adaptive variates of Lasso have been proposed [49, 50]. It is well accepted that Lasso may only be consistent under some situations [47], which is beyond the scope of this study. In this study, we point out that when analyzing SPM and CNV data on the gene level, the collinearity issue is serious given the sparsity of SPM data and overlapped called regions of CNV data. One approach to decrease the collinearity issue in this situation is adding the constraint to require a certain number of mutations in a gene for it to enter the model. For example, filtering the genes with more than 2 mutations before fitting the model would largely decrease the number of genes with high collinearity. Another approach is analyzing on the pathway level instead of gene level, which could largely decrease or avoid collinearity, and meanwhile provide more parsimonious, more stable and more interpretable results.

Our analysis is only based on gene-level data collapsed from variant-level data. Although the consideration of variant-level features is beyond the scope of our study, it is an important consideration and something we hope to explore in future work that evaluates both models estimated on variant-level predictors and alternative approaches for collapsing variant data to gene-level predictors. Although Lasso encounters the instability issue of selecting highly correlated variables, it can be more informative and adaptive to high-dimensional omics data in comparison with ridge [51] and elastic net penalties [52]. In our study, we did not aim at finding the best modeling approach across cancer types. Therefore, the limitation of combining Cox regression models and Lasso penalization exists in our analysis, i.e., the instability issue of selecting highly correlated variables. Ridge regression has been shown to generate more reliable survival predictions than Lasso [51]. It could be combined with pre-filtering procedures to avoid the high dimensionality issue as an alternative to the combination of Cox regression and Lasso [31]. The use of an elastic net penalty (which combines both Lasso and ridge penalties during estimation) is also worth exploring in a pan-cancer setting.

## Conclusions

Our study demonstrates that when using SPM and CNV data for cancer prognosis prediction, pathway-level models are more interpretable, stable and parsimonious compared to gene-level models. Pathway-level models also largely decrease or avoid the issue of collinearity, which can be serious for gene-level somatic alterations data. The prognostic power of somatic alterations is highly variable across different cancer types and we have identified a set of cohorts for which somatic alterations could not predict prognosis. In general, CNV data predicts prognosis better than SPM data with the exception of the LGG cohort.

## Supplementary information

**Additional file 1:** This file contains information on the TCGA cohorts and pathway collections used in this study and supplemental results for both the simulation and real data analyses.

## Data Availability

The datasets analyzed during the current study are available in the UCSC TCGA repository, https://xena.ucsc.edu/, and MSigDB database, https://software.broadinstitute.org/gsea/msigdb/genesets.jsp?collection=H.

## Abbreviations

TCGA: The Cancer Genome Atlas
SPM: Somatic point mutation
CNV: Copy number variation
TMB: Tumor mutation burden
GSE: Gene set enrichment
MSigDB: Molecular Signatures Database
CAMERA: Correlation Adjusted Mean Rank gene set test
GSEA: Gene set enrichment analysis
GSVA: Gene Set Variation Analysis
PLAGE: Pathway Level Analysis of Gene Expression
MC3: Multi-center Mutation Calling in Multiple Cancers
CI: Concordance index
