# Supplementary material for: Cancer prognosis prediction using somatic point mutation and copy number variation data: a comparison of gene-level and pathway-based models
Source: BMC Bioinformatics. 2020 Oct 20;21:467. doi: 10.1186/s12859-020-03791-0 (PMC7574407; doi:10.1186/s12859-020-03791-0)

Cancer prognosis prediction using somatic point mutation and copy number variation data: a comparison of gene-level and pathway-based models

Xingyu Zheng, Christopher I. Amos, H. Robert Frost

List of Contents:

List of Tables ………………………………………………………………………………… 3

X1 The full list of 29 cohorts analyzed in this study and some clinical characteristics. ……… 3
X2 Detailed information about pathway collections used in this study. ………………………….. 4
X3 The predictors retained by the pathway-level LGG SPM model using binary method and HALLMARK collection. ……………………………………………………………………………… 4
X4 The predictors retained by the pathway-level LGG SPM model using log OR method and HALLMARK collection. ……………………………………………………………………………… 4

List of Figures ………………………………………………………………………………. 5

S1 The results of null models. ………………………………………………………………………… 5
S2-S30 The distribution of concordance index values for all models estimated on all cohorts. .. 7
S2 ACC cohort ……………………………………………………………………………… 8
S3 BLCA cohort …………………………………………………………………………… 9
S4 BRCA cohort …………………………………………………………………………… 10
S5 CESC cohort …………………………………………………………………………… 11
S6 COAD cohort ………………………………………………………………………….. 12
S7 ESCA cohort …………………………………………………………………………… 13
S8 GBM cohort ……………………………………………………………………………. 14
S9 HNSC cohort ………………………………………………………………………….. 15
S10 KICH cohort ……………………………………………………………………………. 16
S11 KIRC cohort ……………………………………………………………………………. 17
S12 KIRP cohort ……………………………………………………………………………. 18
S13 LAML cohort …………………………………………………………………………… 19
S14 LGG cohort …………………………………………………………………………….. 20

S15 LIHC cohort 21
S16 LUAD cohort 22
S17 LUSC cohort 23
S18 MESO cohort 24
S19 OV cohort 25
S20 PAAD cohort 26
S21 PCPG cohort 27
S22 PRAD cohort 28
S23 READ cohort 29
S24 SARC cohort 30
S25 STAD cohort 31
S26 TGCT cohort 32
S27 THCA cohort 33
S28 THYM cohort 34
S29 UCEC cohort 35
S30 UVM cohort 36

Results of simulation study for Lasso 37

S31 Solution path of Lasso 38

Table X1 The full list of 29 cohorts analyzed in this study and some clinical characteristics.

Cohort	Full name	Sample size CNV	Sample size SPM	Death rate CNV	Death rate SPM
ACC	Adrenocortical Carcinoma	89	90	0.36	0.37
BLCA	Bladder Carcinoma	404	391	0.44	0.45
BRCA	Breast Cancer	1064	759	0.14	0.15
CESC	Cervical Cancer	282	196	0.24	0.21
COAD	Colon Cancer	428	210	0.22	0.23
ESCA	Esophageal Cancer	184	183	0.41	0.41
GBM	Glioblastoma	570	277	0.81	0.75
HNSC	Head and Neck Cancer	519	507	0.42	0.43
KICH	Kidney Chromophobe	65	65	0.14	0.14
KIRC	Kidney Clear Cell Carcinoma	526	213	0.33	0.25
KIRP	Kidney Papillary Cell Carcinoma	285	279	0.15	0.15
LAML	Acute Myeloid Leukemia	166	171	0.63	0.62
LGG	Lower Grade Glioma	508	511	0.25	0.24
LIHC	Liver Cancer	364	199	0.35	0.45
LUAD	Lung Adenocarcinoma	503	477	0.36	0.36
LUSC	Lung Squamous Cell Carcinoma	494	175	0.43	0.49
MESO	Mesothelioma	85	81	0.86	0.85
OV	Ovarian Cancer	564	141	0.59	0.65
PAAD	Pancreatic Cancer	183	183	0.55	0.54
PCPG	Pheochromocytoma and Paraganglioma	160	177	0.04	0.03
PRAD	Prostate Cancer	491	497	0.02	0.02
READ	Rectal Cancer	157	80	0.17	0.20
SARC	Sarcoma	257	255	0.38	0.38
STAD	Stomach Cancer	413	355	0.40	0.37
TGCT	Testicular Cancer	134	134	0.03	0.03
THCA	Thyroid Cancer	498	495	0.03	0.03
THYM	Thymoma	122	122	0.07	0.07
UCEC	Endometroid Cancer	537	194	0.17	0.13
UVM	Ocular Melanoma	80	80	0.29	0.29

Table X2 Detailed information about pathway collections used in this study.

	Pathway size	Subset of	Derivation source
HALLMARK	50	HALLMARK	Condense pathways from all collections in MSigDB
PID	196	C2CP	Pathway Interaction Database
BIOCARTA	289	C2CP	BioCarta pathway database
REACTOME	1499	C2CP	Reactome pathway database
BP	1350	C5	GO Biological Process Ontology

Table X3 The predictors retained by LASSO for the pathway-level LGG SPM model where predictor values were generated using binary method for the pathways in the MSigDB HALLMARK collection.

	coef	pvalue
HALLMARK_APICAL_JUNCTION	0.84	4.13E-05
HALLMARK_PROTEIN_SECRETION	0.79	1.74E-04
HALLMARK_REACTIVE_OXIGEN_SPECIES_PATHWAY	-1.40	3.30E-03
HALLMARK_BILE_ACID_METABOLISM	-1.35	4.54E-03
HALLMARK_MTORC1_SIGNALING	-0.36	0.23
HALLMARK_OXIDATIVE_PHOSPHORYLATION	-0.07	0.87

Table X4 The predictors retained by LASSO for the pathway-level LGG SPM model where predictor values were generated using log-odds ratios for the pathways in the MSigDB HALLMARK collection.

	coef	pvalue
HALLMARK_PROTEIN_SECRETION	0.70	2.74E-06
HALLMARK_APICAL_JUNCTION	0.35	7.22E-03
HALLMARK_BILE_ACID_METABOLISM	-0.53	0.06
HALLMARK_MTORC1_SIGNALING	-0.31	0.12
HALLMARK_OXIDATIVE_PHOSPHORYLATION	-0.16	0.54
HALLMARK_XENOBIOTIC_METABOLISM	-0.07	0.74

The results of null models

To ensure the predictive signals are not generated randomly and that the prediction is not inflated in our analyses, we checked the results of null models, in which all the steps are the same except that the survival outcomes are shuffled among individuals to break any association between the variables and the outcomes, while maintain the correlations among variables.

Figure S1 Heatmap of the concordance index for different null models and cancer types. “PLv” represents “Pathway-level” and “GLv” represents “Gene-level”. HALLMARK, PID, BIOCARTA, REACTOME and BP are the used five pathway collections as introduced in Methods. Corresponding to each model introduced in Methods: “log OR” and “Binary” represent the two enrichment methods for SPM data; “all genes”, “Path intersected”, “Cosmic intersected”, “Cox filter” represents the filters on the genes. The maximum value among all is 0.59, the minimum is 0.46 and the average is 0.50.

The results of these null models show that for all the models and cohorts, the concordance is around the expected null value of 0.5, which demonstrates that the signals in our true models are valid.

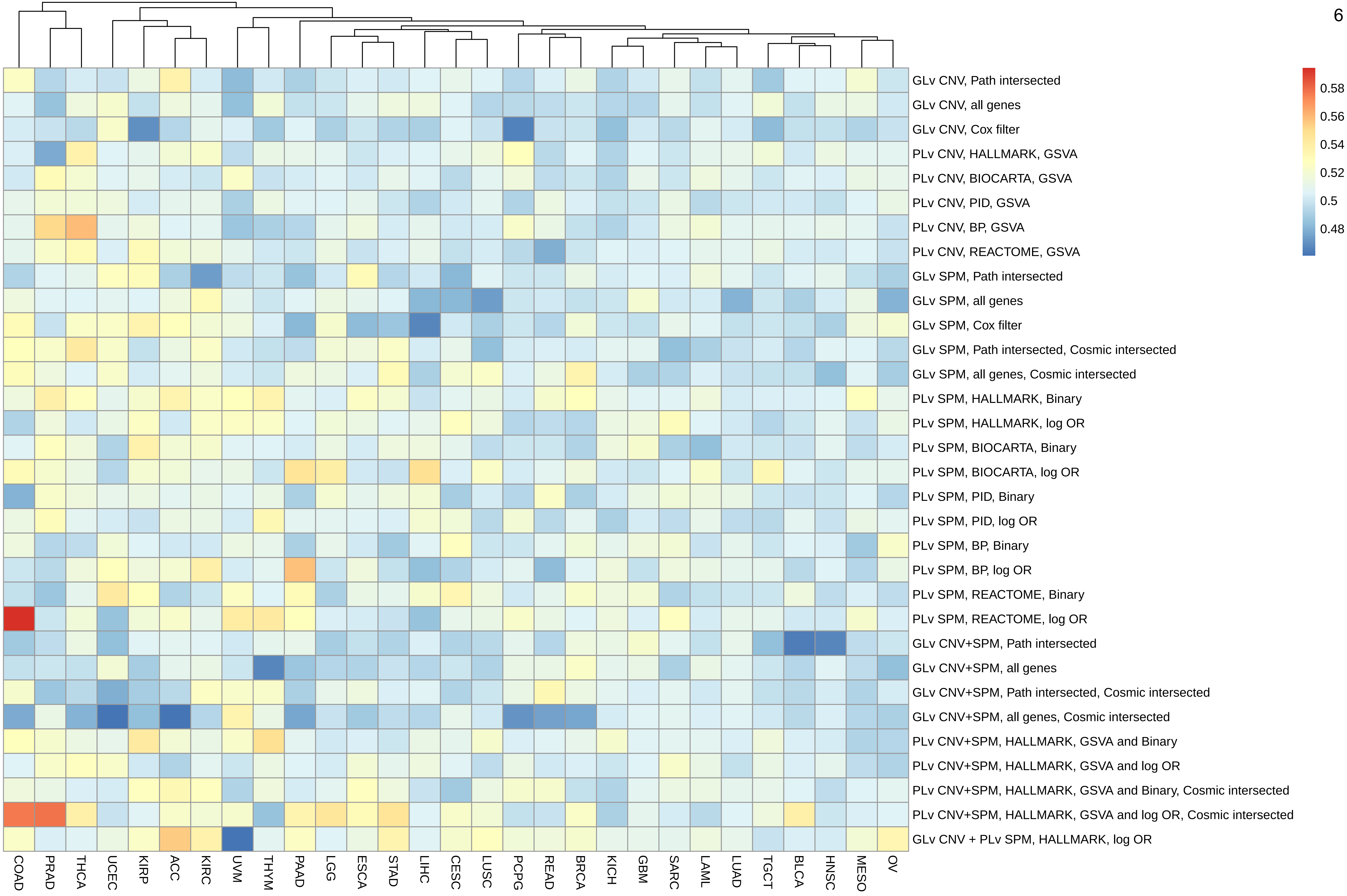
GLv CNV, Path intersected
GLv CNV, all genes
GLv CNV, Cox filter
PLv CNV, HALLMARK, GSVA
PLv CNV, BIOCARTA, GSVA
PLv CNV, PID, GSVA
PLv CNV, BP, GSVA
PLv CNV, REACTOME, GSVA
GLv SPM, Path intersected
GLv SPM, all genes
GLv SPM, Cox filter
GLv SPM, Path intersected, Cosmic intersected
GLv SPM, all genes, Cosmic intersected
PLv SPM, HALLMARK, Binary
PLv SPM, HALLMARK, log OR
PLv SPM, BIOCARTA, Binary
PLv SPM, BIOCARTA, log OR
PLv SPM, PID, Binary
PLv SPM, PID, log OR
PLv SPM, BP, Binary
PLv SPM, BP, log OR
PLv SPM, REACTOME, Binary
PLv SPM, REACTOME, log OR
GLv CNV+SPM, Path intersected
GLv CNV+SPM, all genes
GLv CNV+SPM, Path intersected, Cosmic intersected
GLv CNV+SPM, all genes, Cosmic intersected
PLv CNV+SPM, HALLMARK, GSVA and Binary
PLv CNV+SPM, HALLMARK, GSVA and log OR
PLv CNV+SPM, HALLMARK, GSVA and Binary, Cosmic intersected
PLv CNV+SPM, HALLMARK, GSVA and log OR, Cosmic intersected
GLv CNV + PLv SPM, HALLMARK, log OR
COAD
PRAD
THCA
UCEC
KIRP
ACC
KIRC
UVM
THYM
PAAD
LGG
ESCA
STAD
LIHC
CESC
LUSC
PCPG
READ
BRCA
KICH
GBM
SARC
LAML
LUAD
TGCT
BLCA
HNSC
MESO
OV
0.58
0.56
0.54
0.52
0.5
0.48

The results of the distribution of concordance index values for all models estimated on all cohorts.

Figure S2-S30 Distribution of concordance index values for all models evaluated on each cohort. "PLv" represents "Pathway-level" and "GLv" represents "Gene-level". HALLMARK, PID, BIOCARTA, REACTOME and BP are the used five pathway collections as introduced in Methods. Corresponding to each model introduced in Methods: "log OR" and "Binary" represent the two enrichment methods for SPM data; "all genes", "Path intersected", "Cosmic intersected", "Cox filter" represents the filters on the genes.

Figure S2 ACC cohort

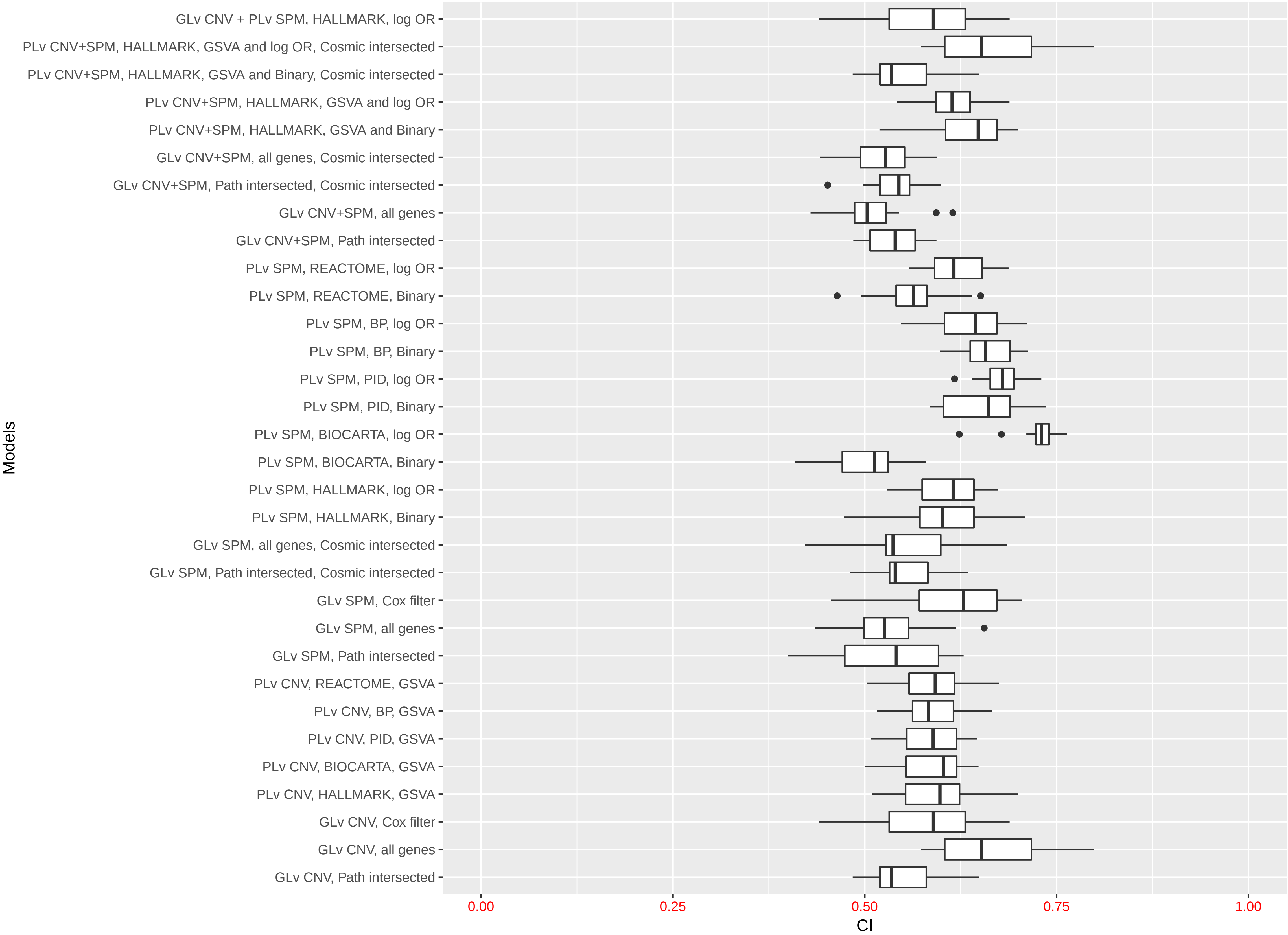

Figure S3 BLCA cohort

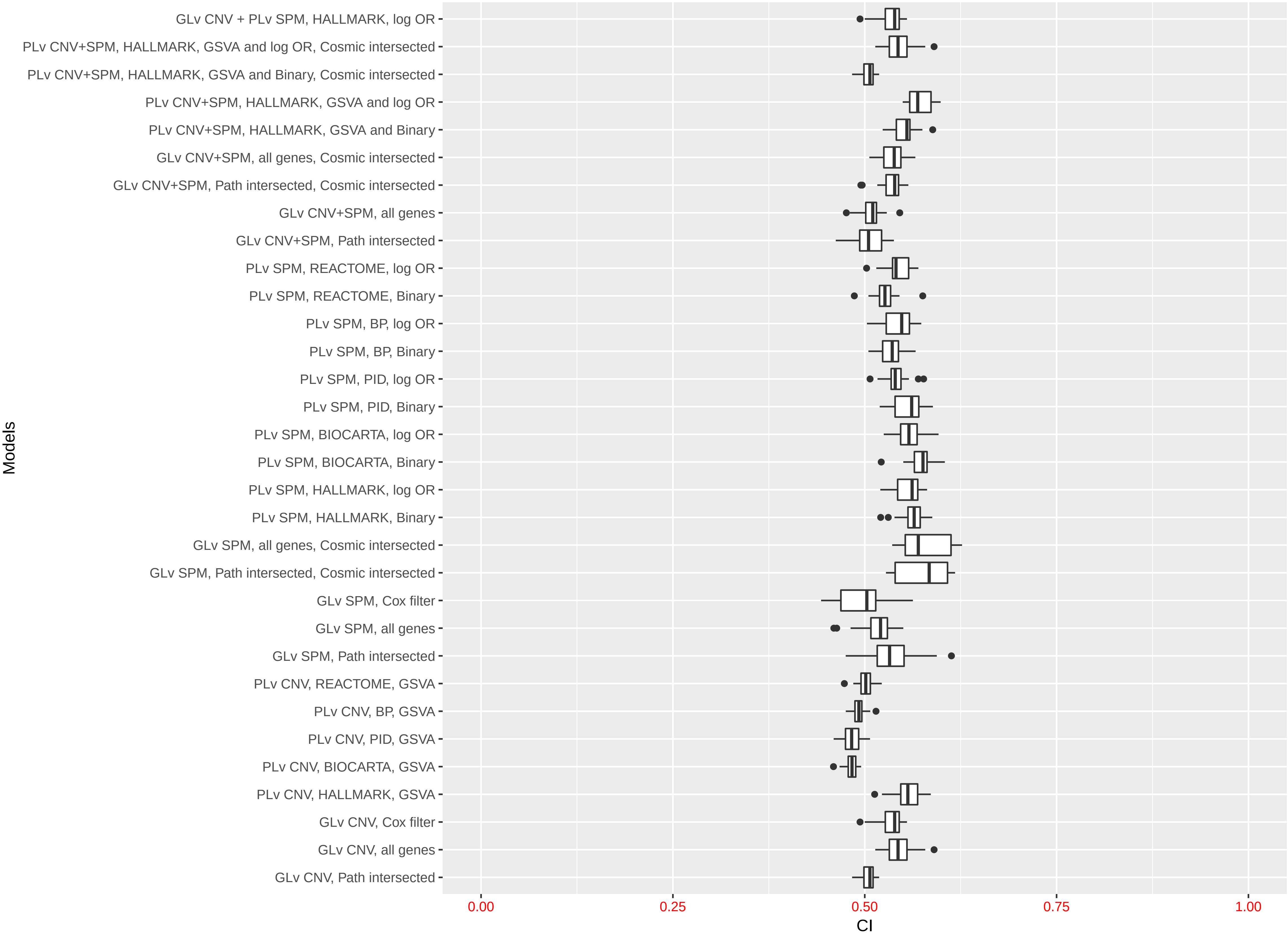

Figure S4 BRCA cohort

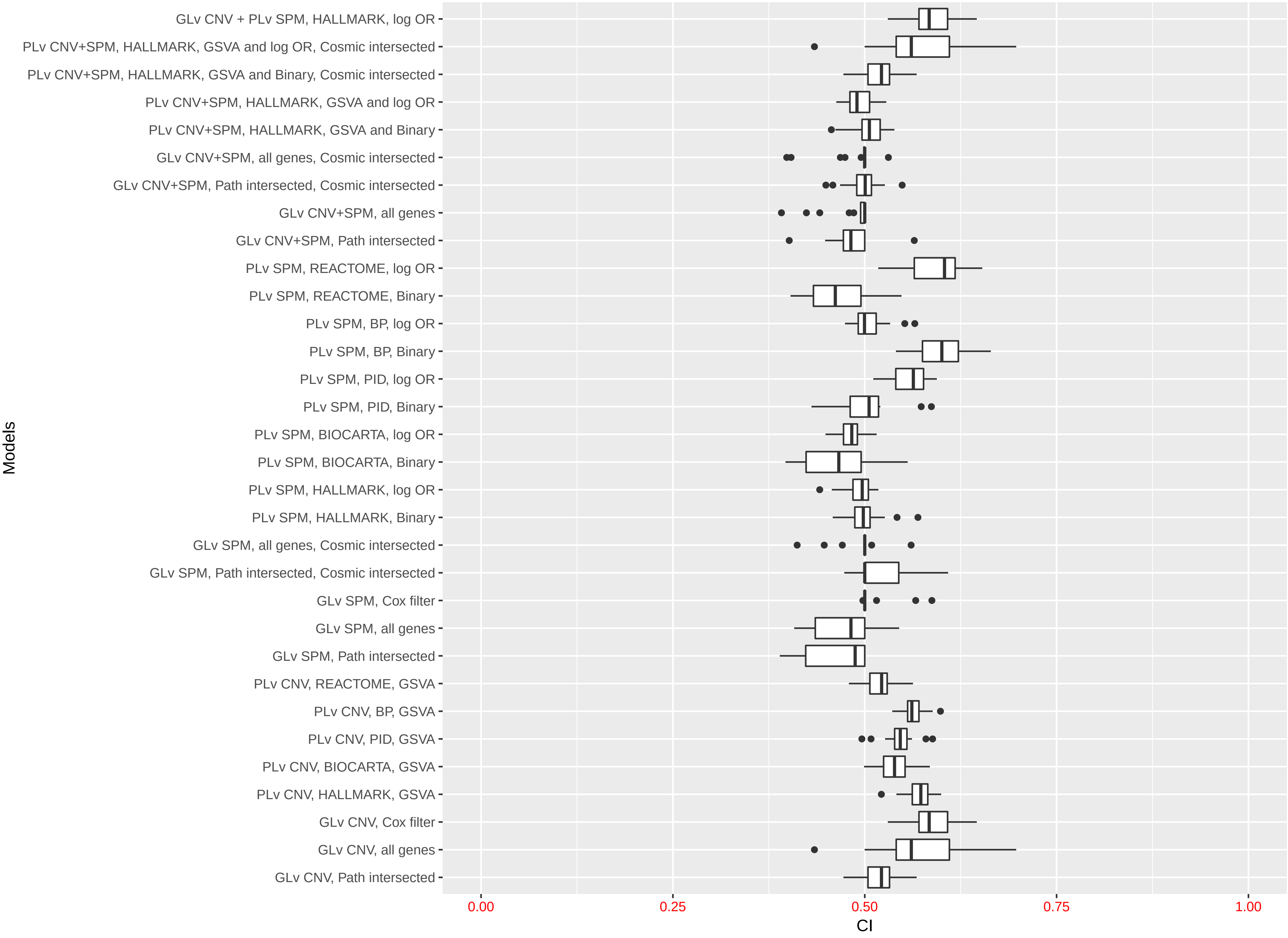

Figure S5 CESC cohort

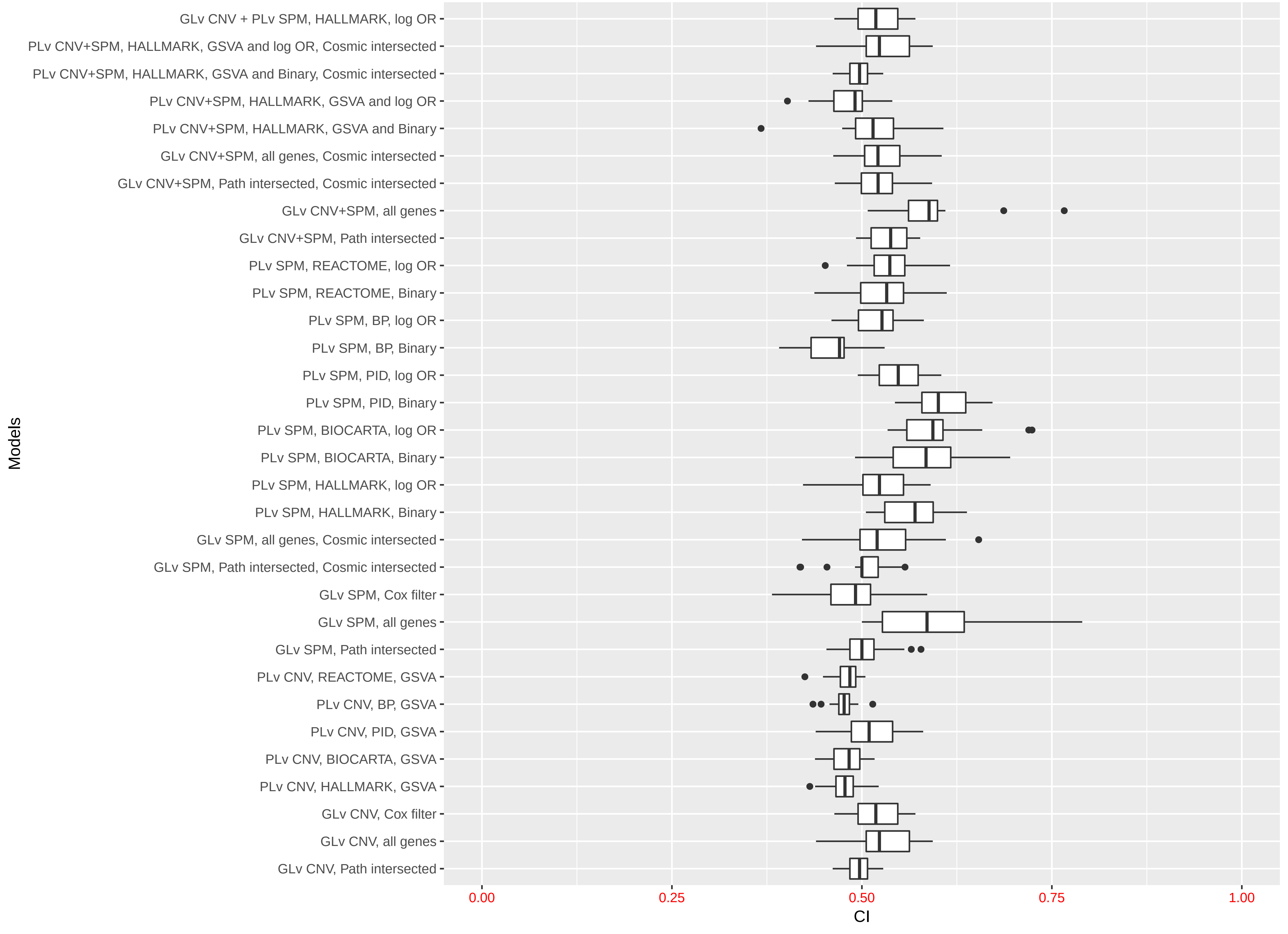

Figure S6 COAD cohort

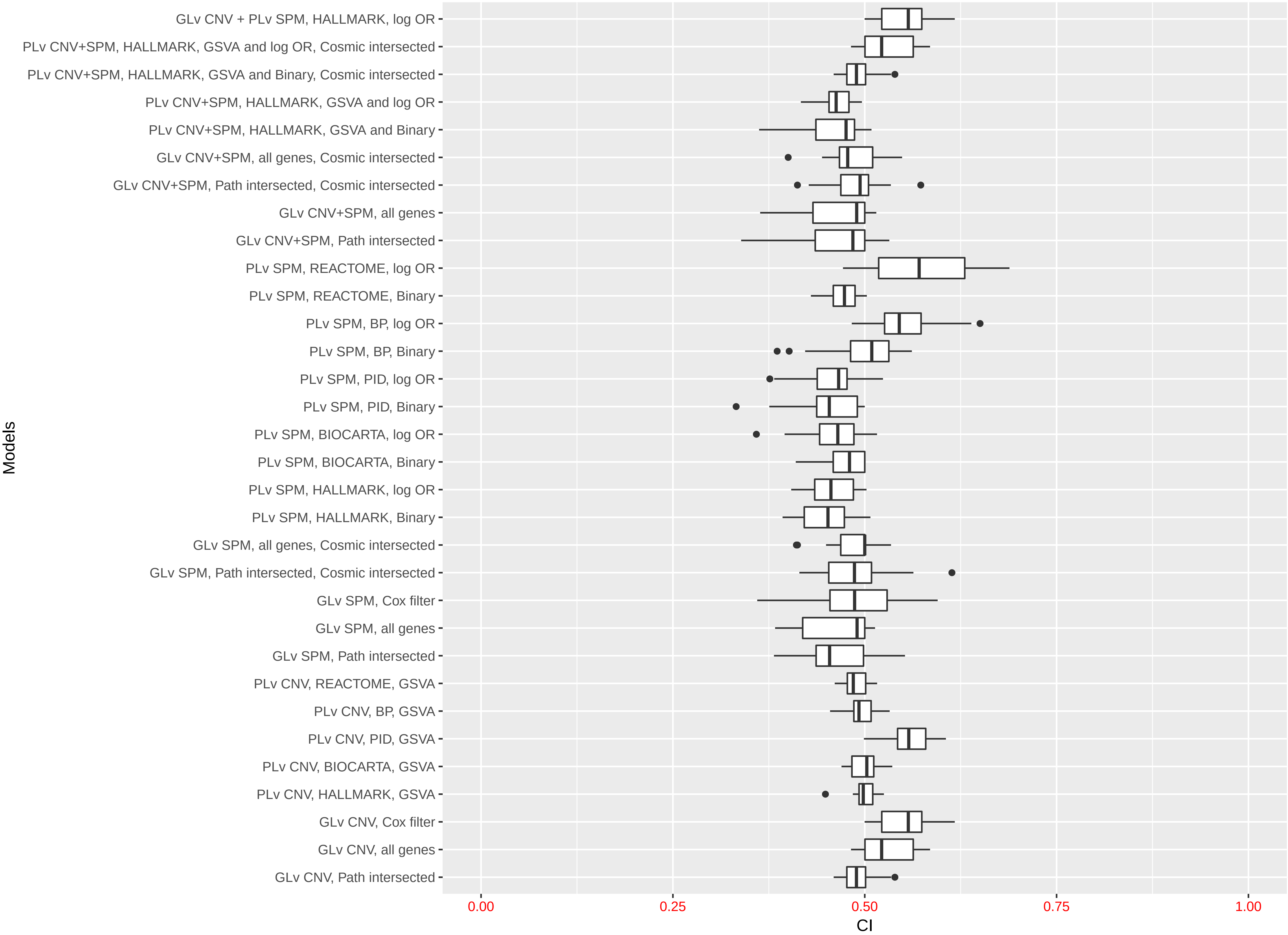

Figure S7 ESCA cohort

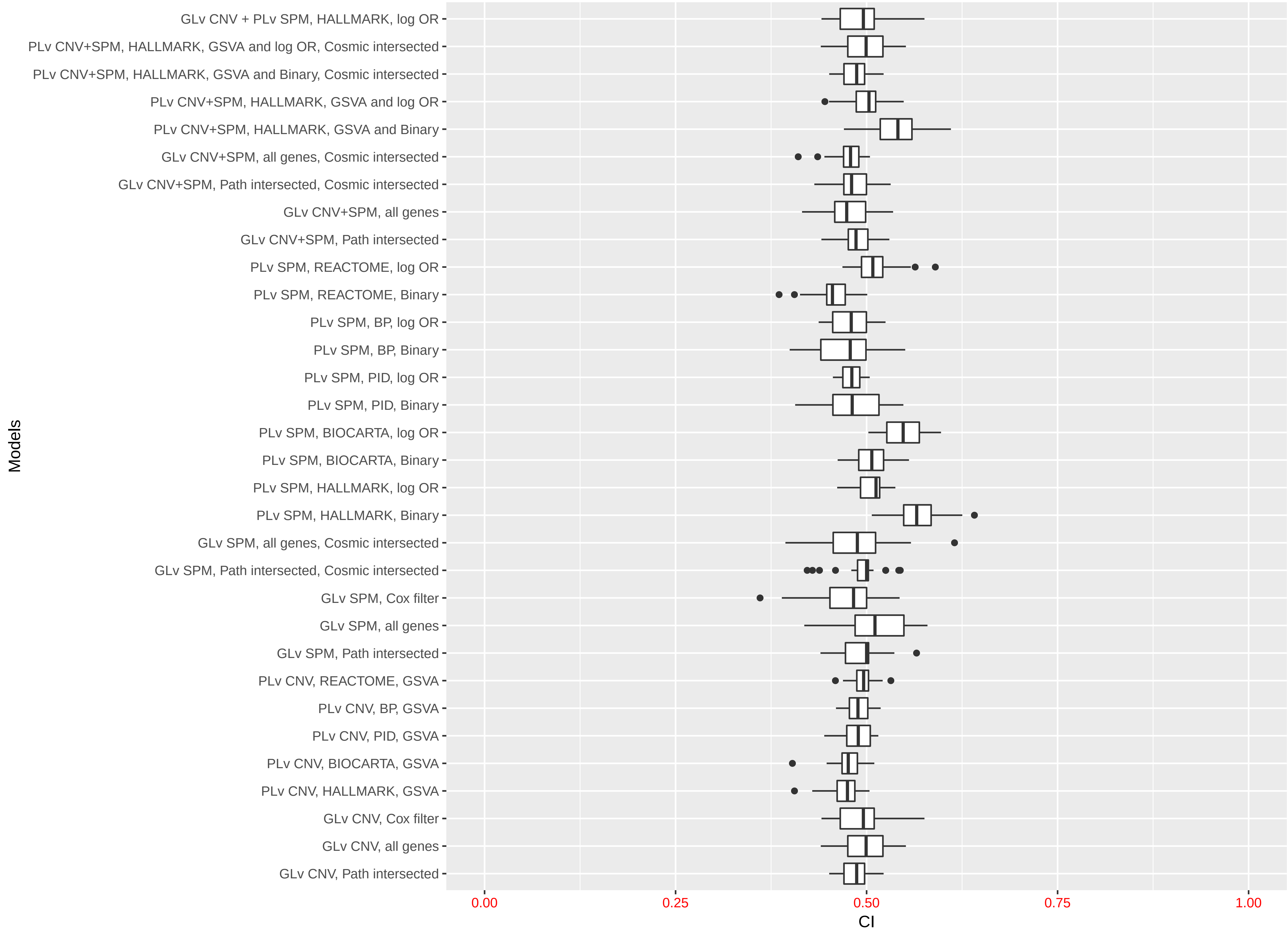

Figure S8 GBM cohort

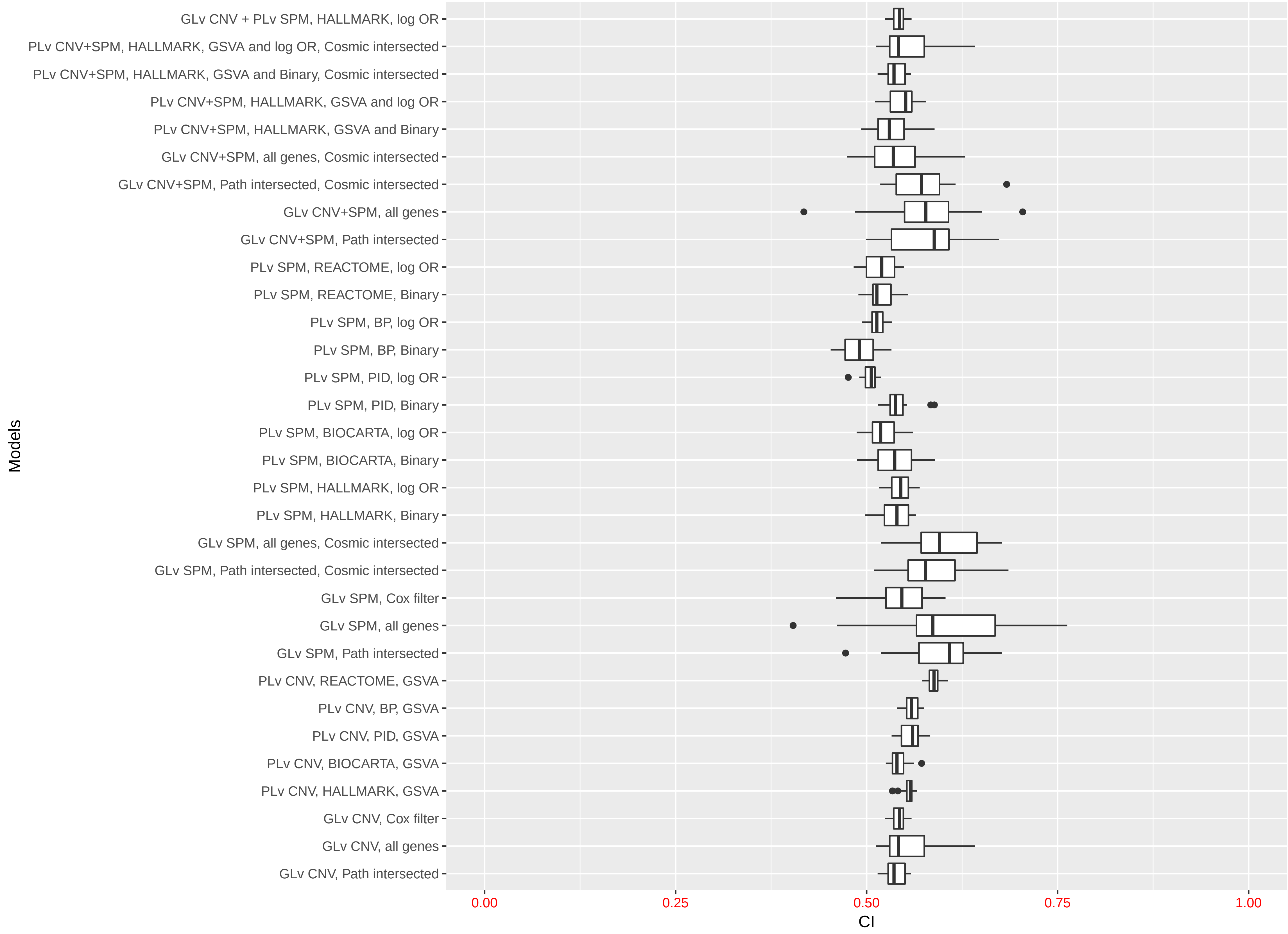

Figure S9 HNSC cohort

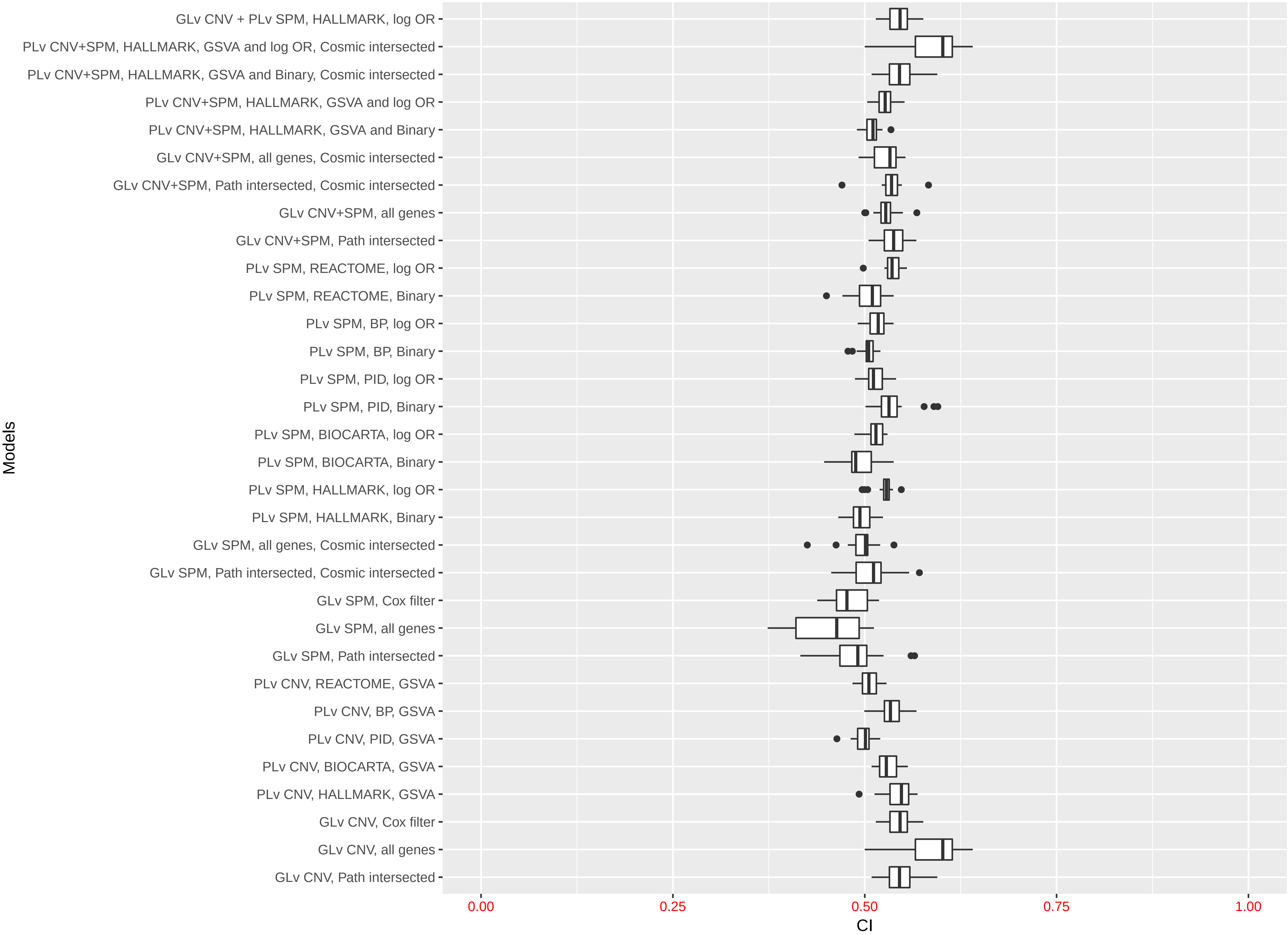

Figure S10 KICH cohort

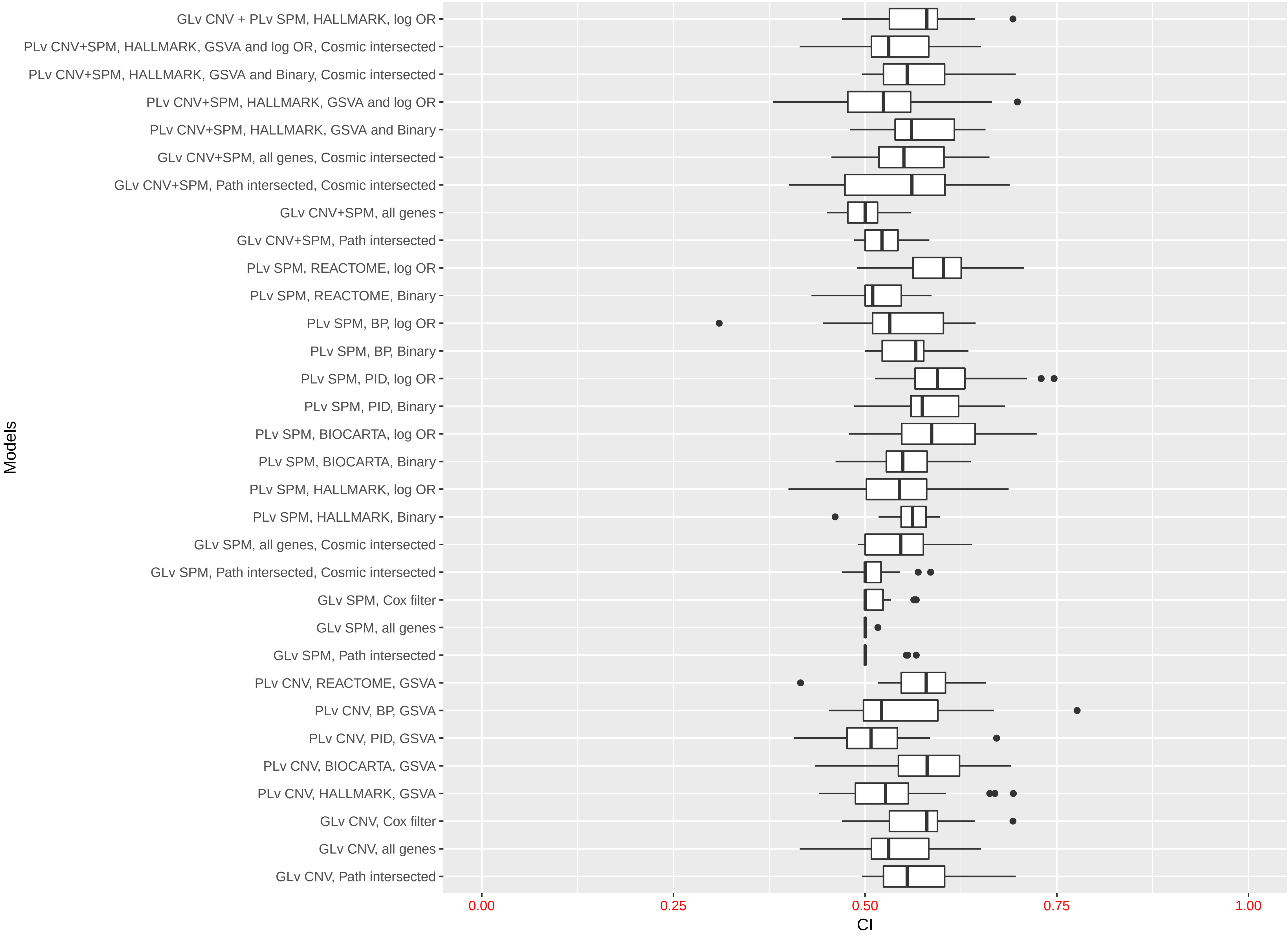

Figure S11 KIRC cohort

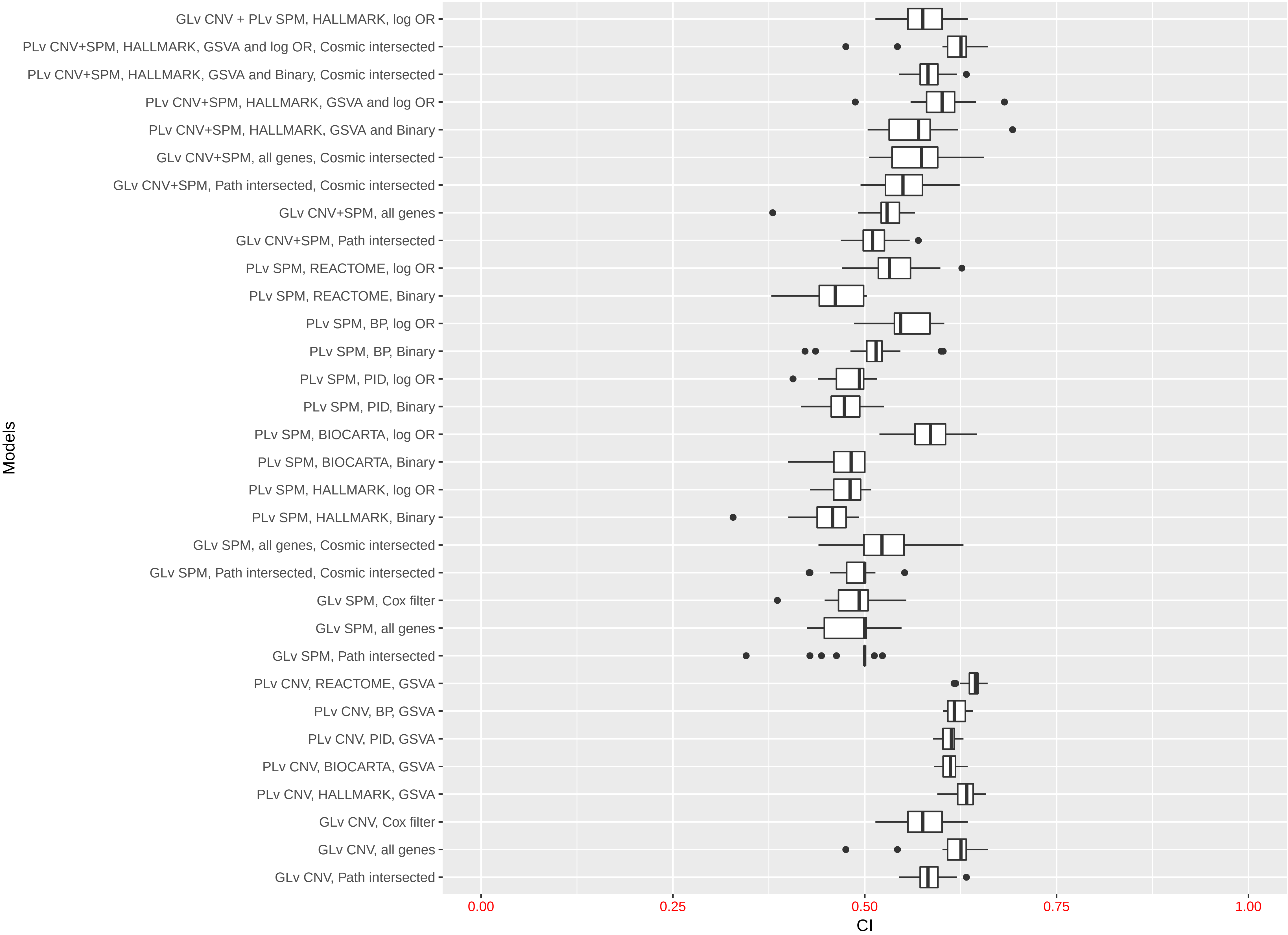

Figure S12 KIRP cohort

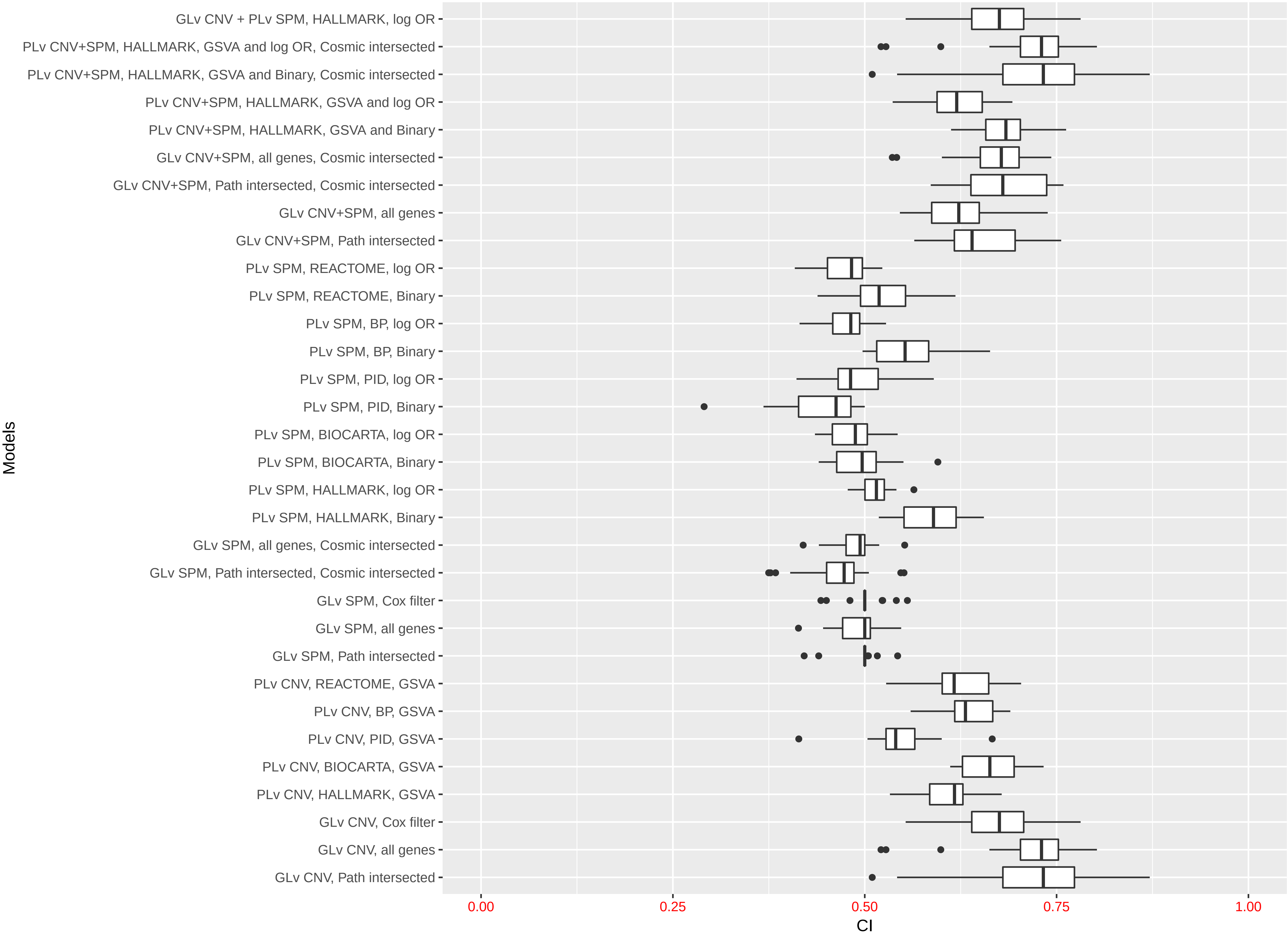

Figure S13 LAML cohort

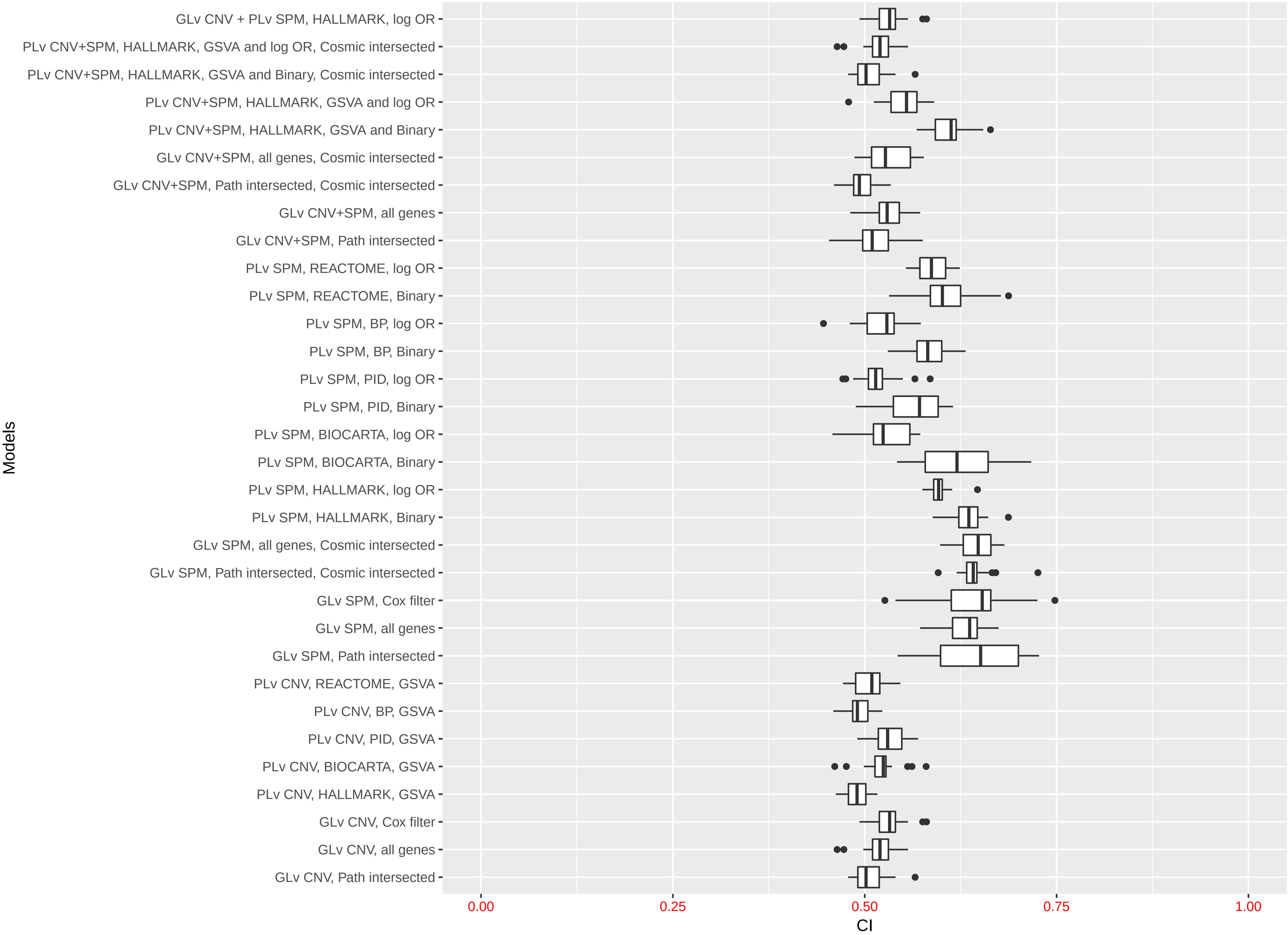

Figure S14 LGG cohort

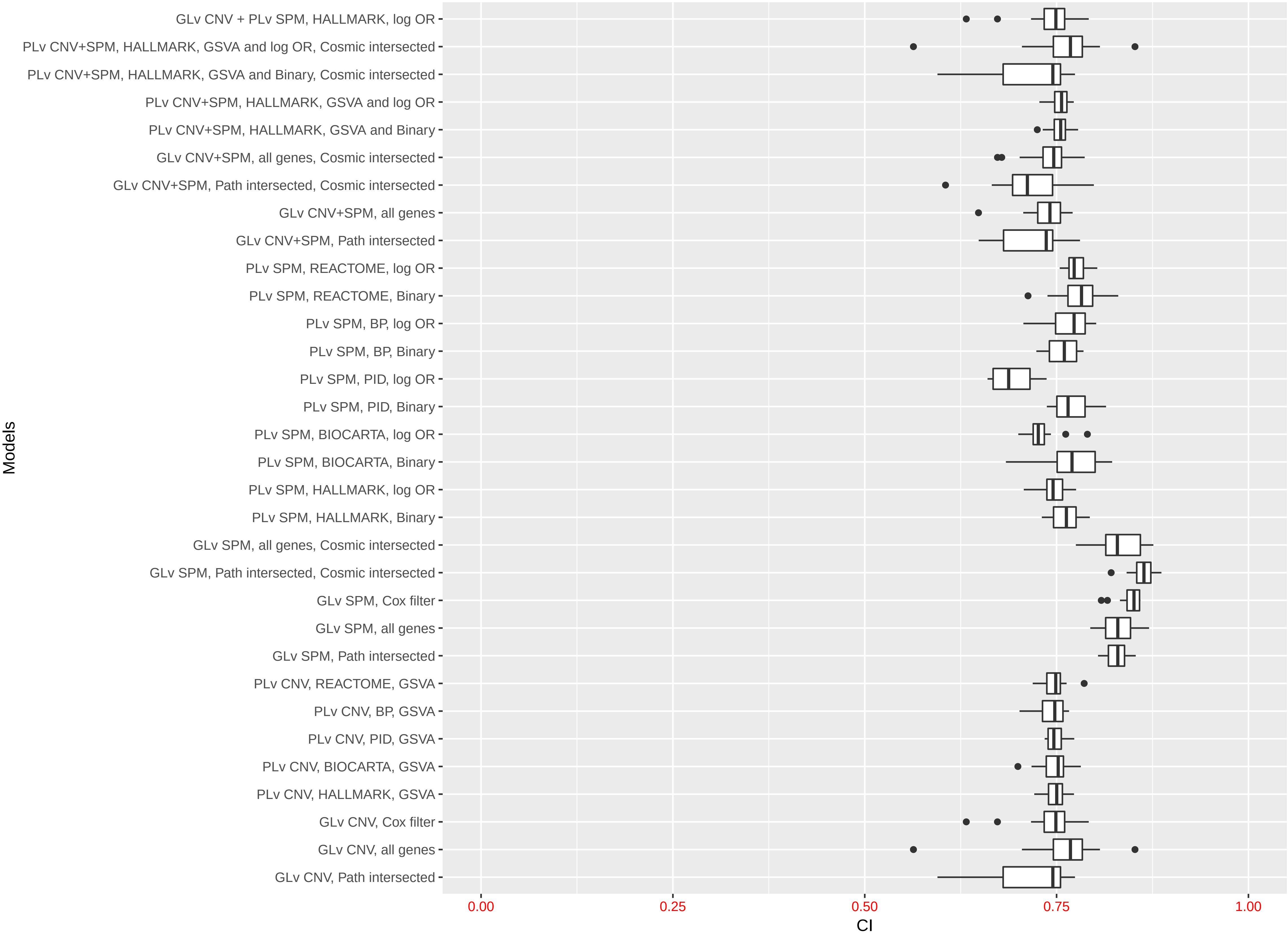

Figure S15 LIHC cohort

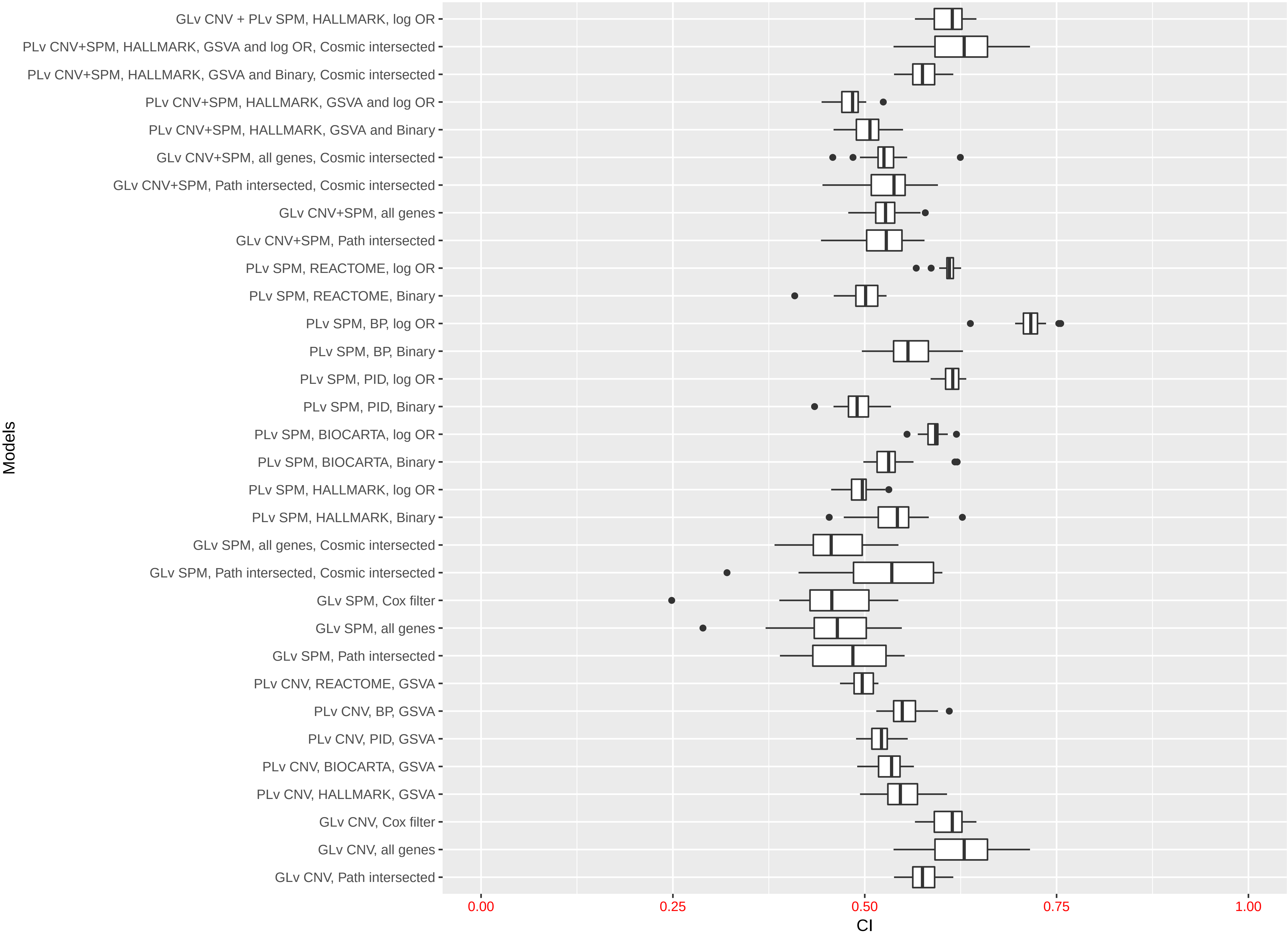

Figure S16 LUAD cohort

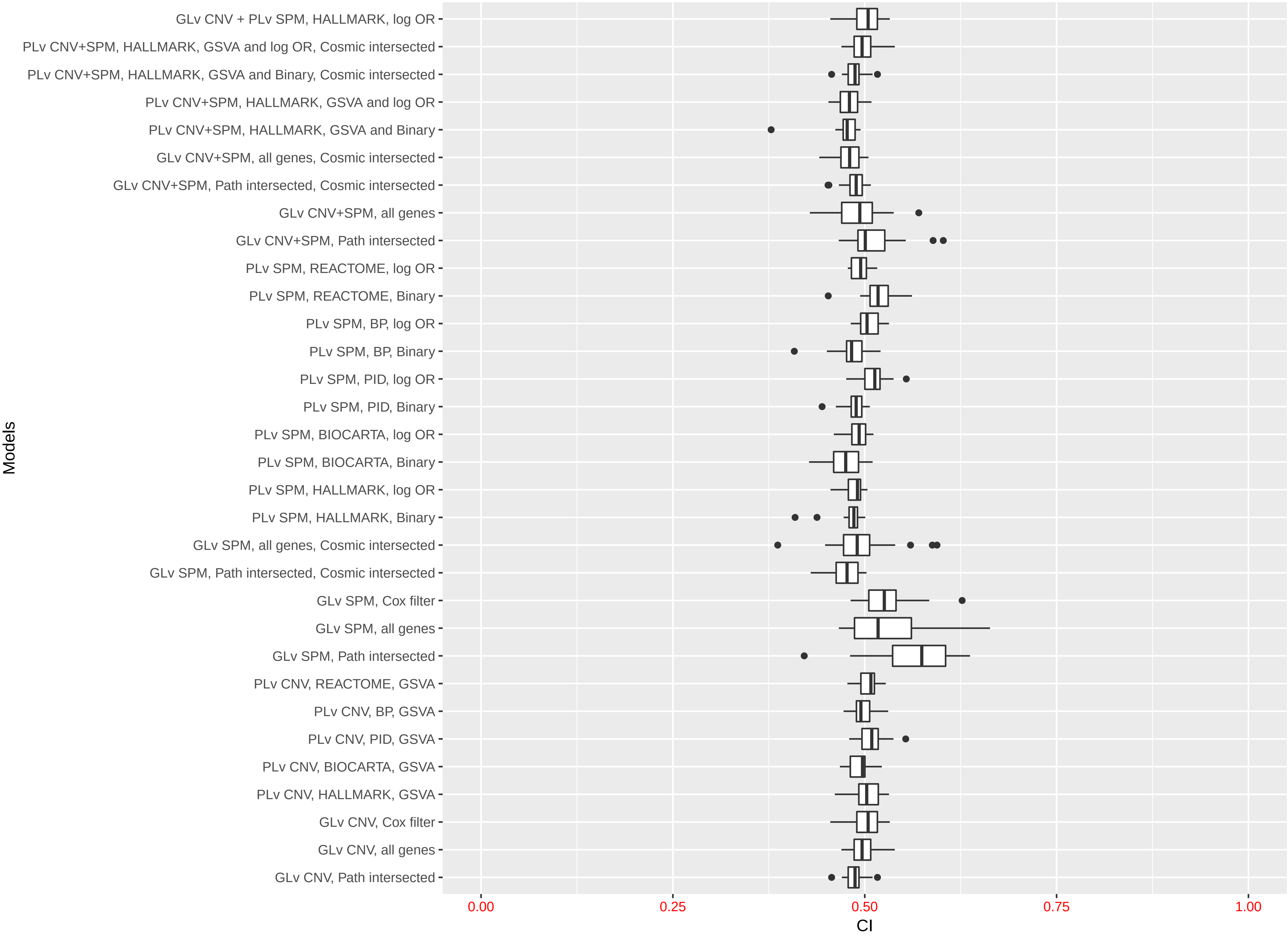

Figure S17 LUSC cohort

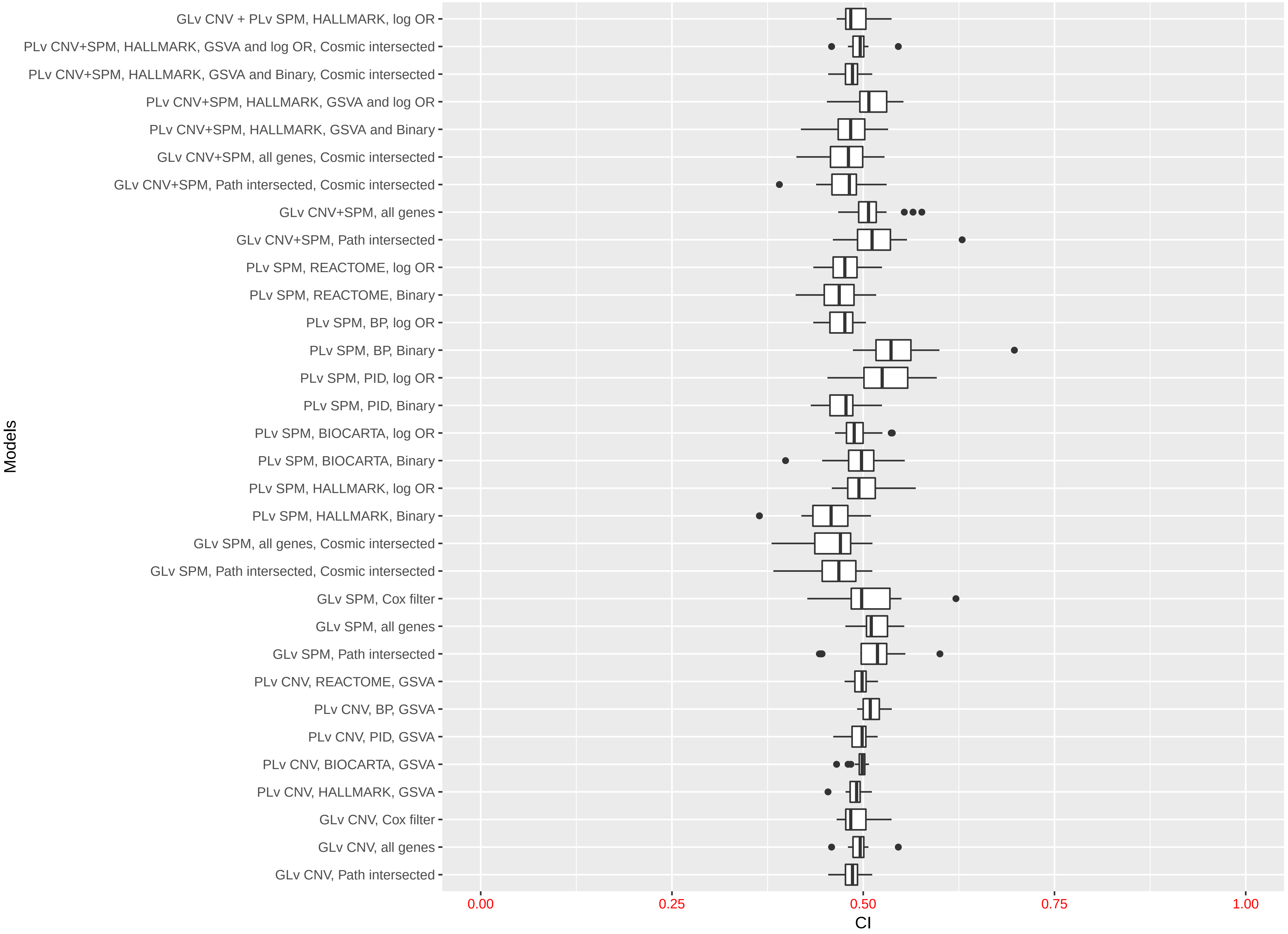

Figure S18 MESO cohort

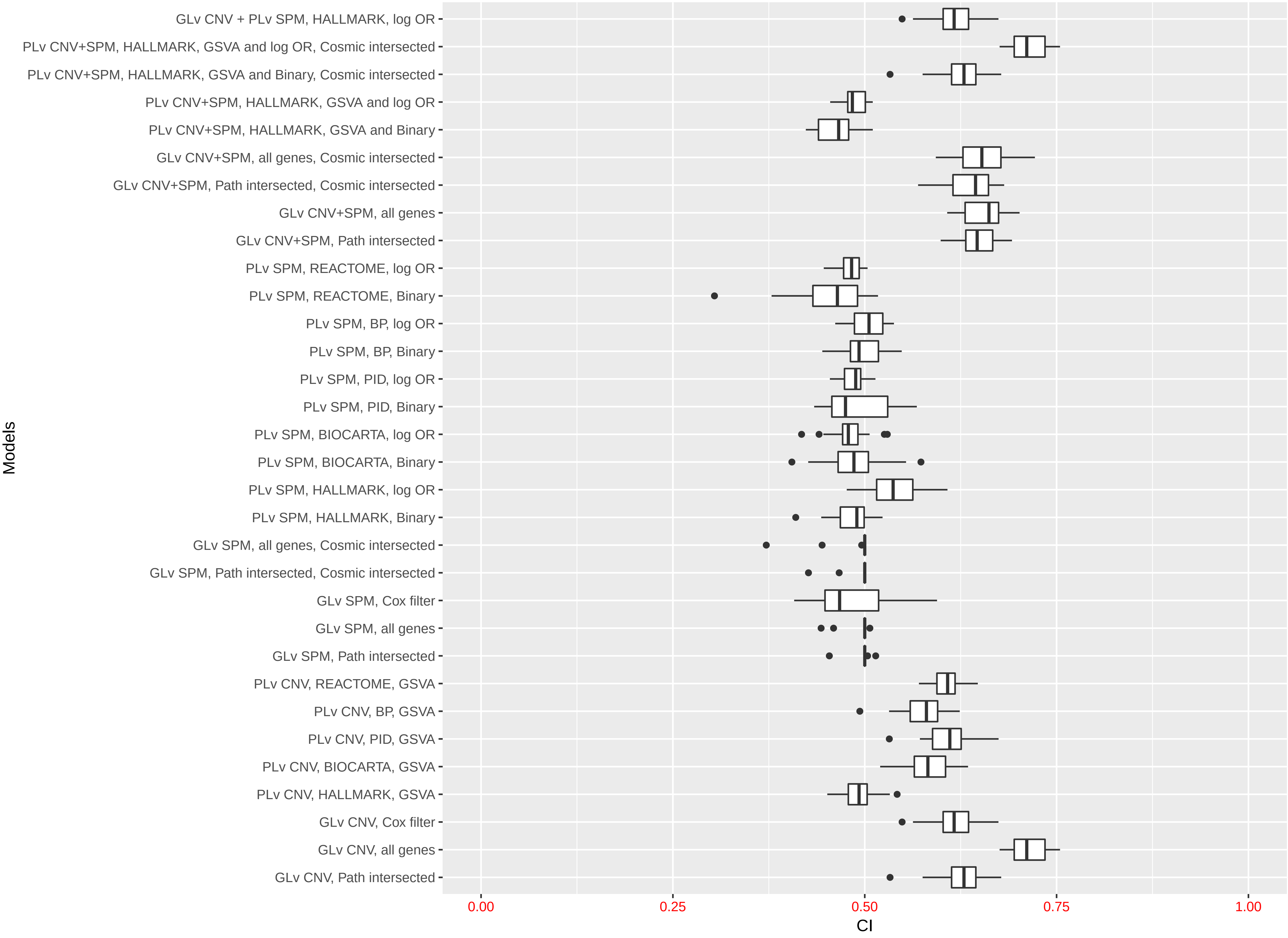

Figure S19 OV cohort

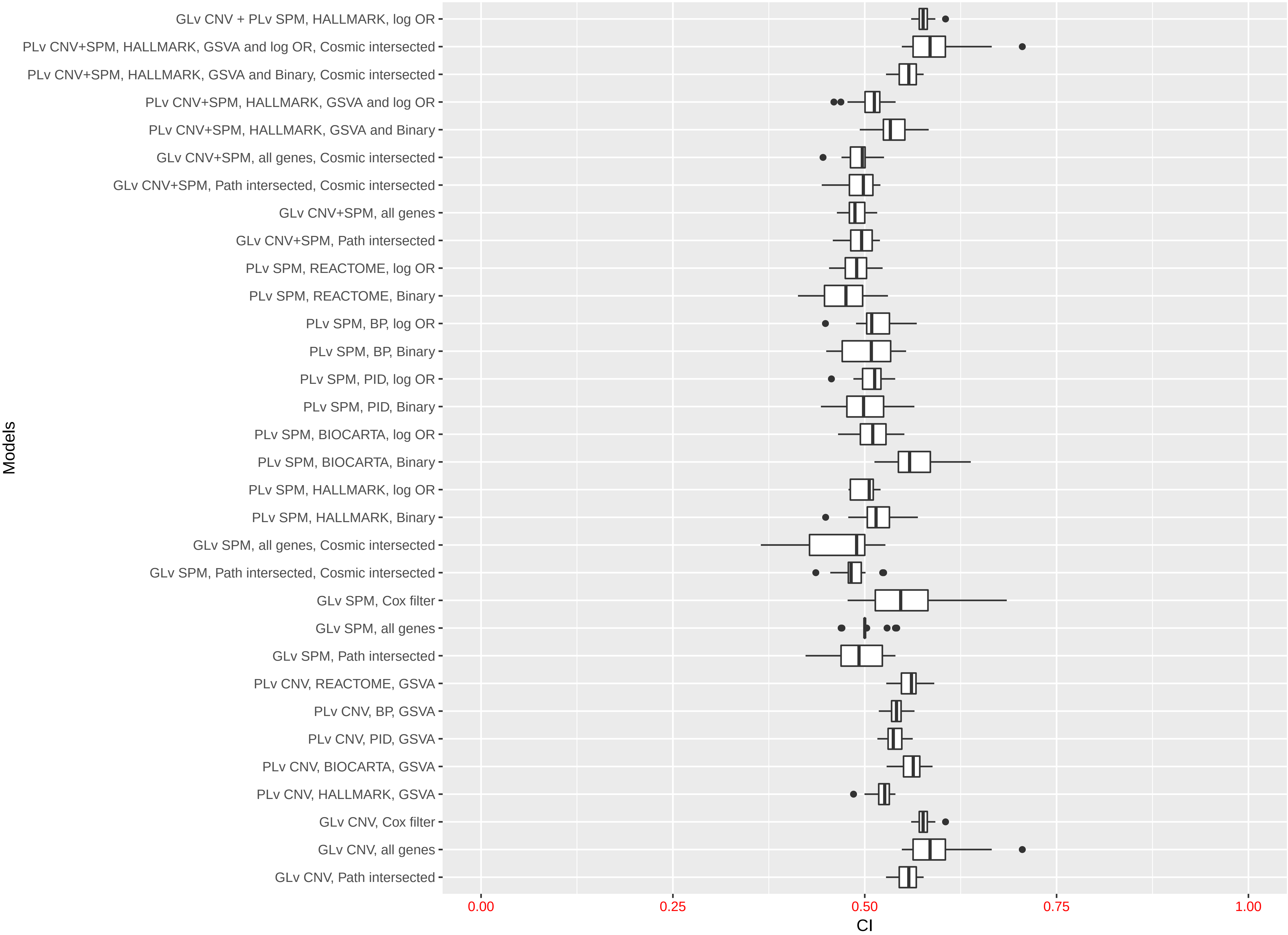

Figure S20 PAAD cohort

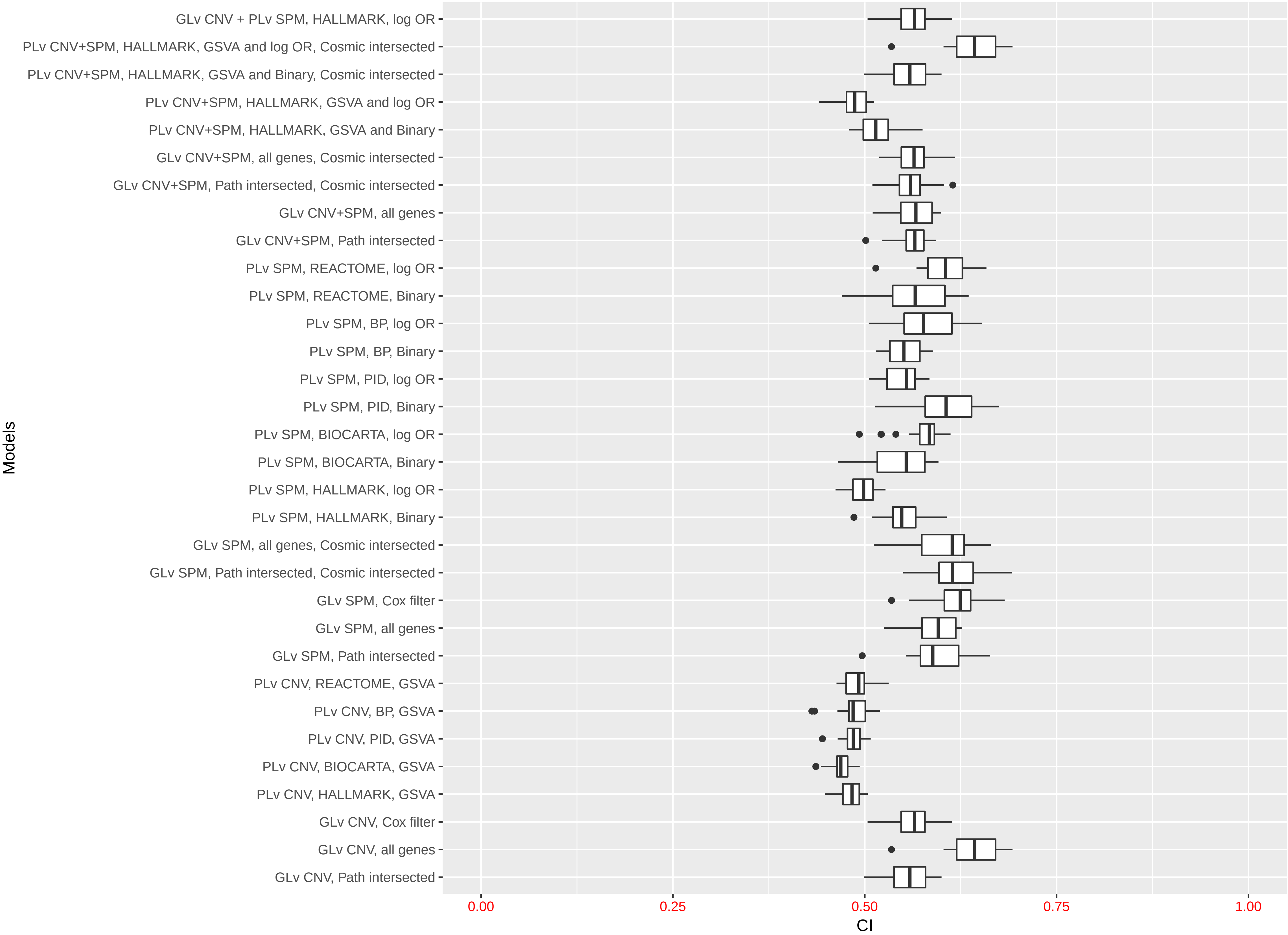

Figure S21 PCPG cohort

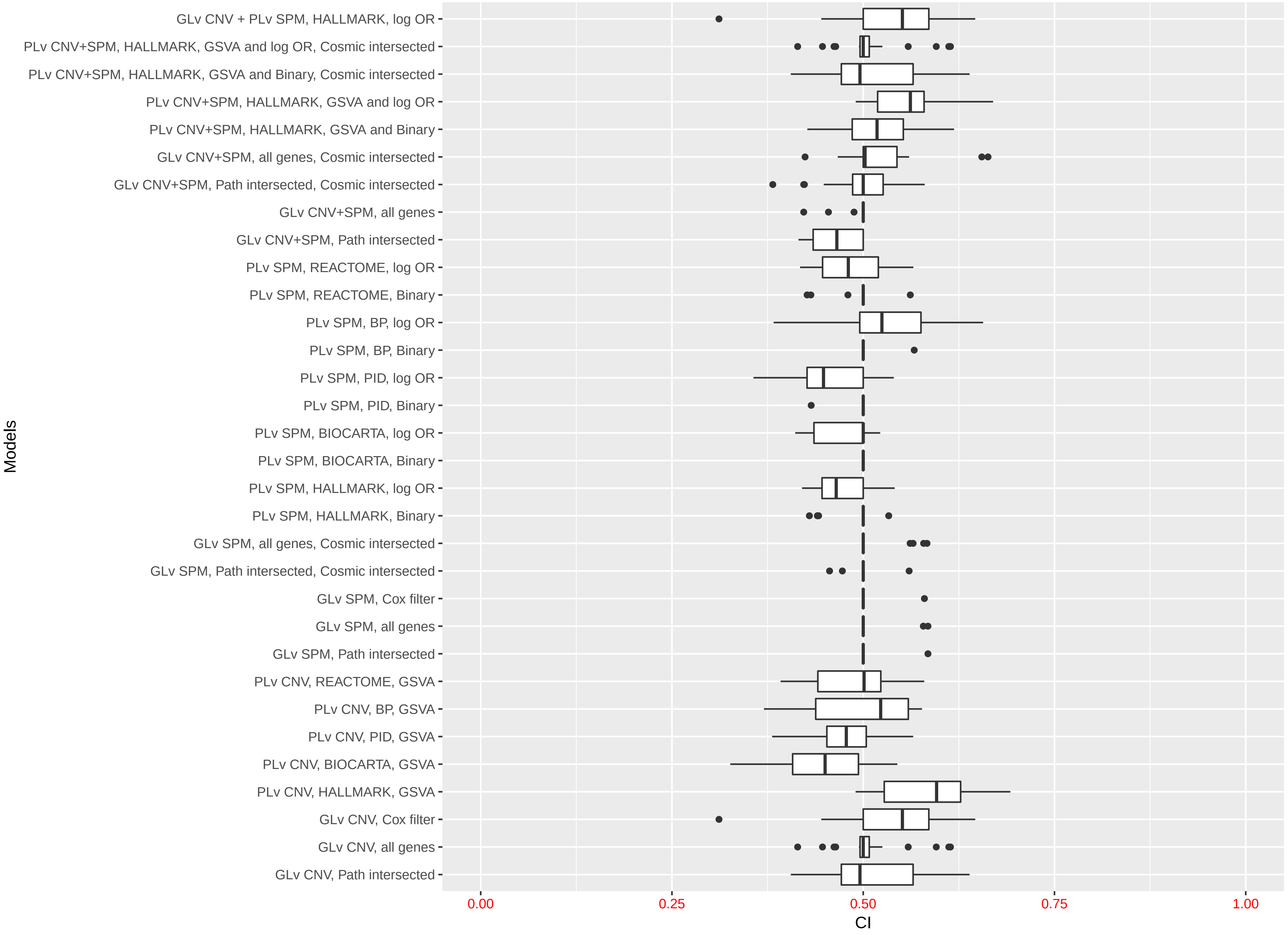

Figure S22 PRAD cohort

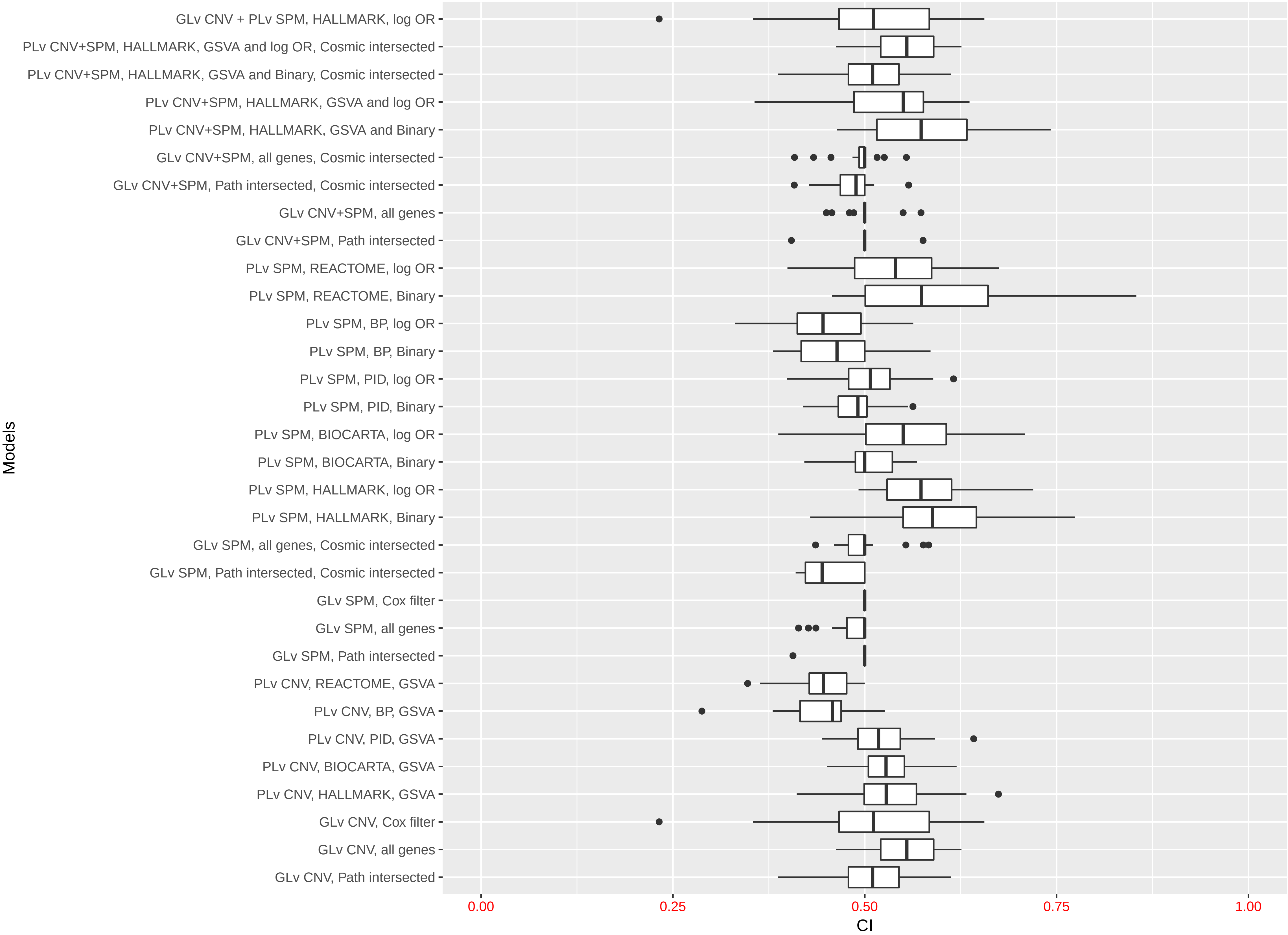

Figure S23 READ cohort

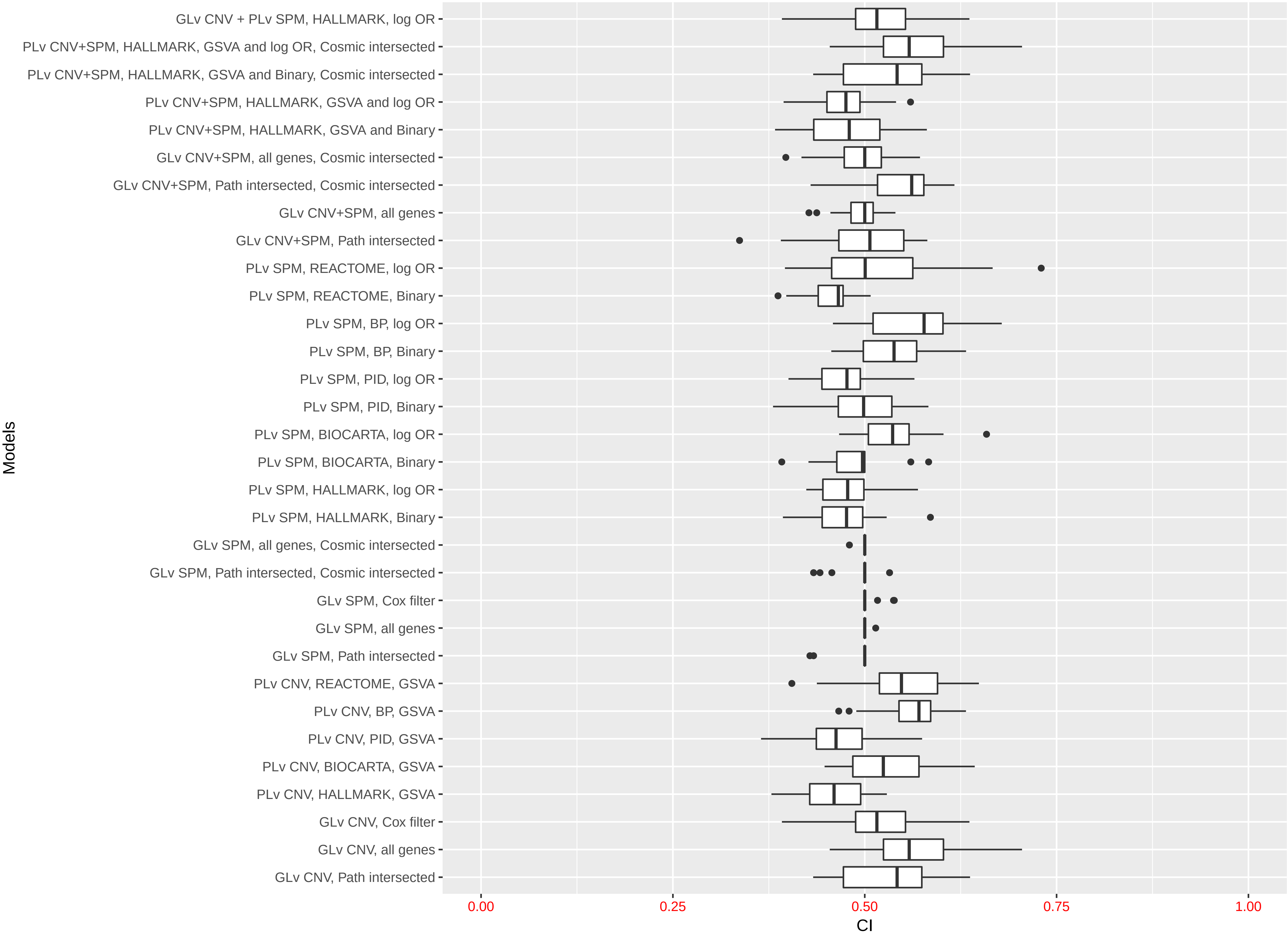

Figure S24 SARC cohort

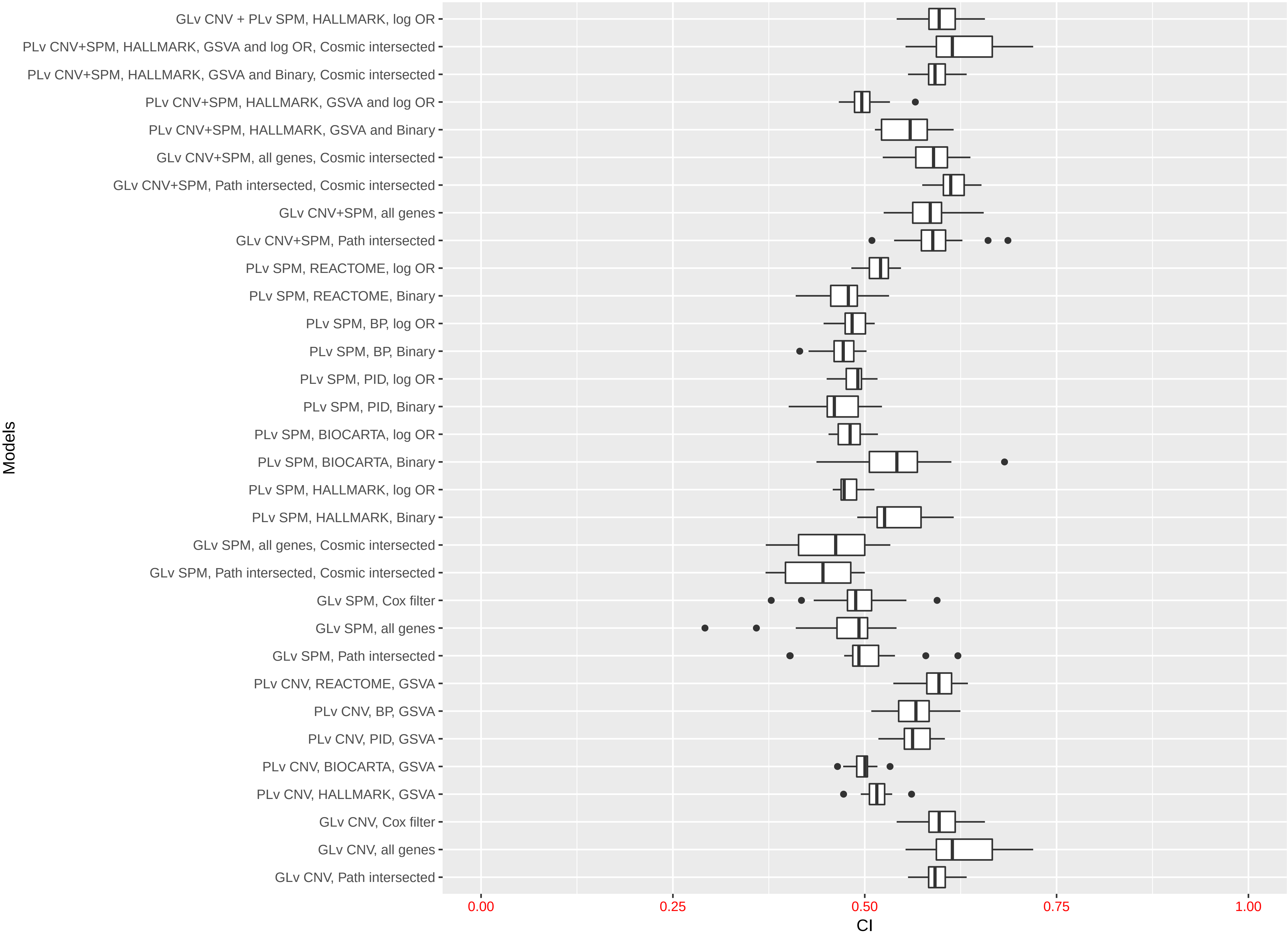

Figure S25 STAD cohort

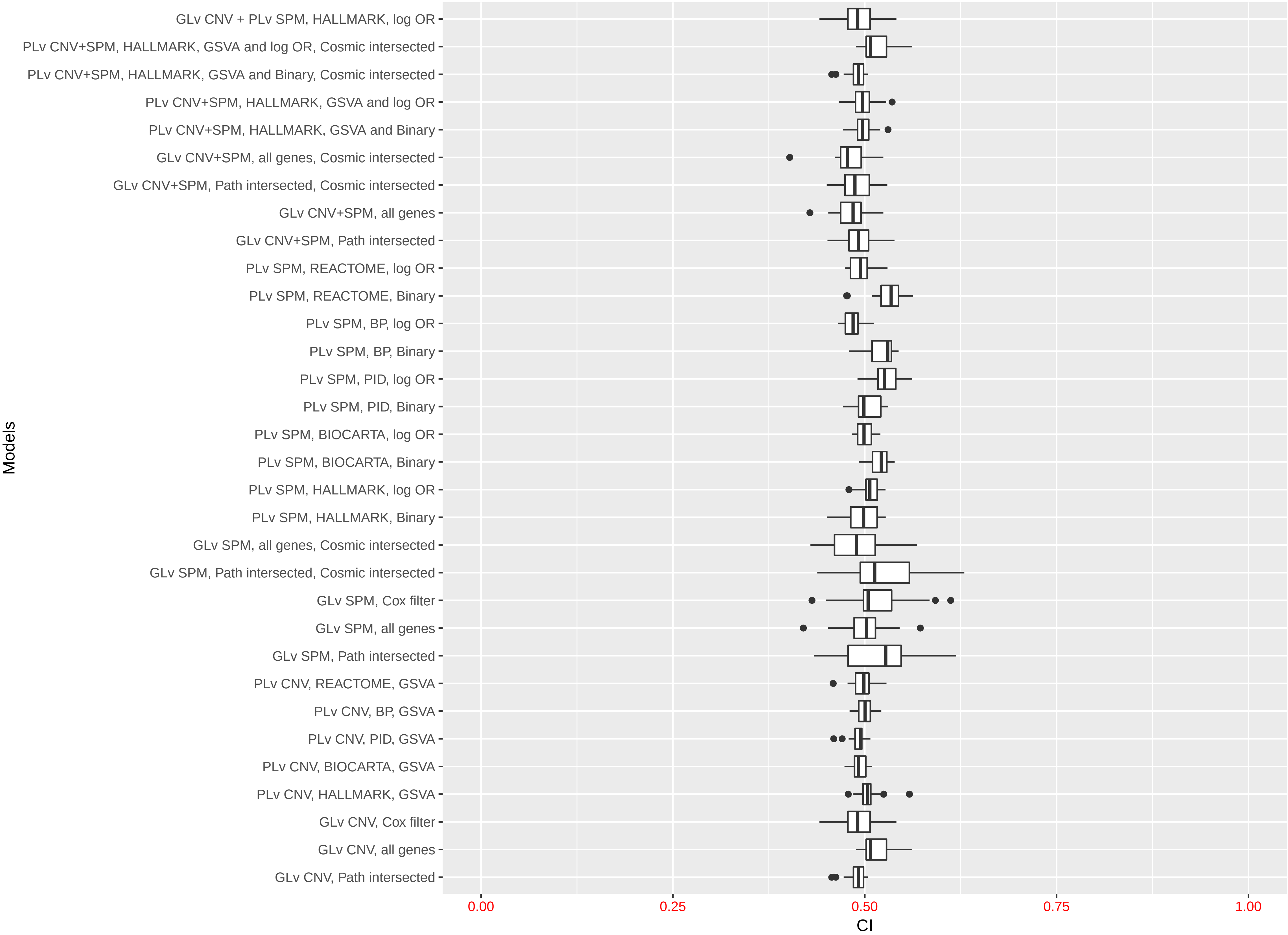

Figure S26 TGCT cohort

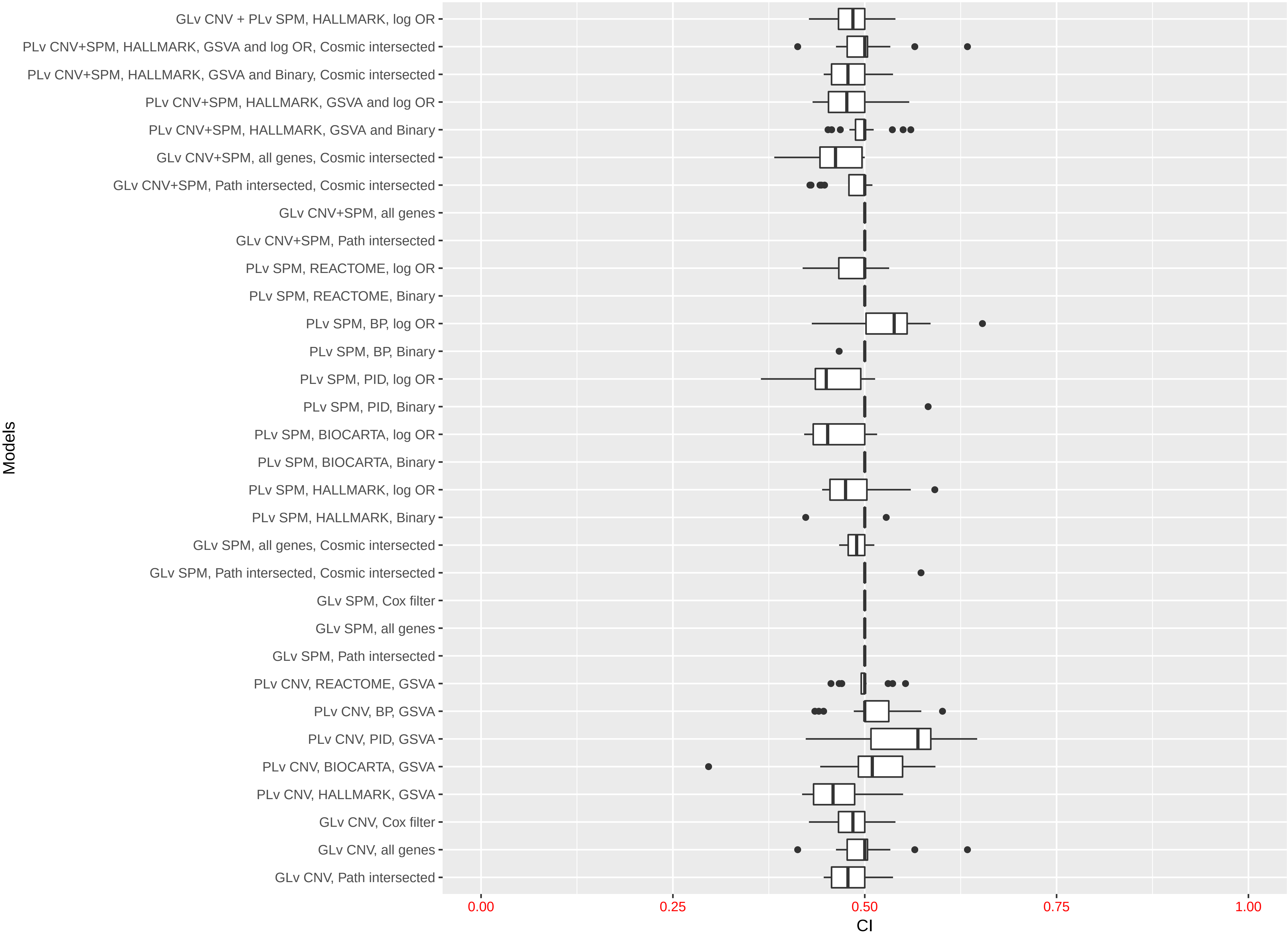

Figure S27 THCA cohort

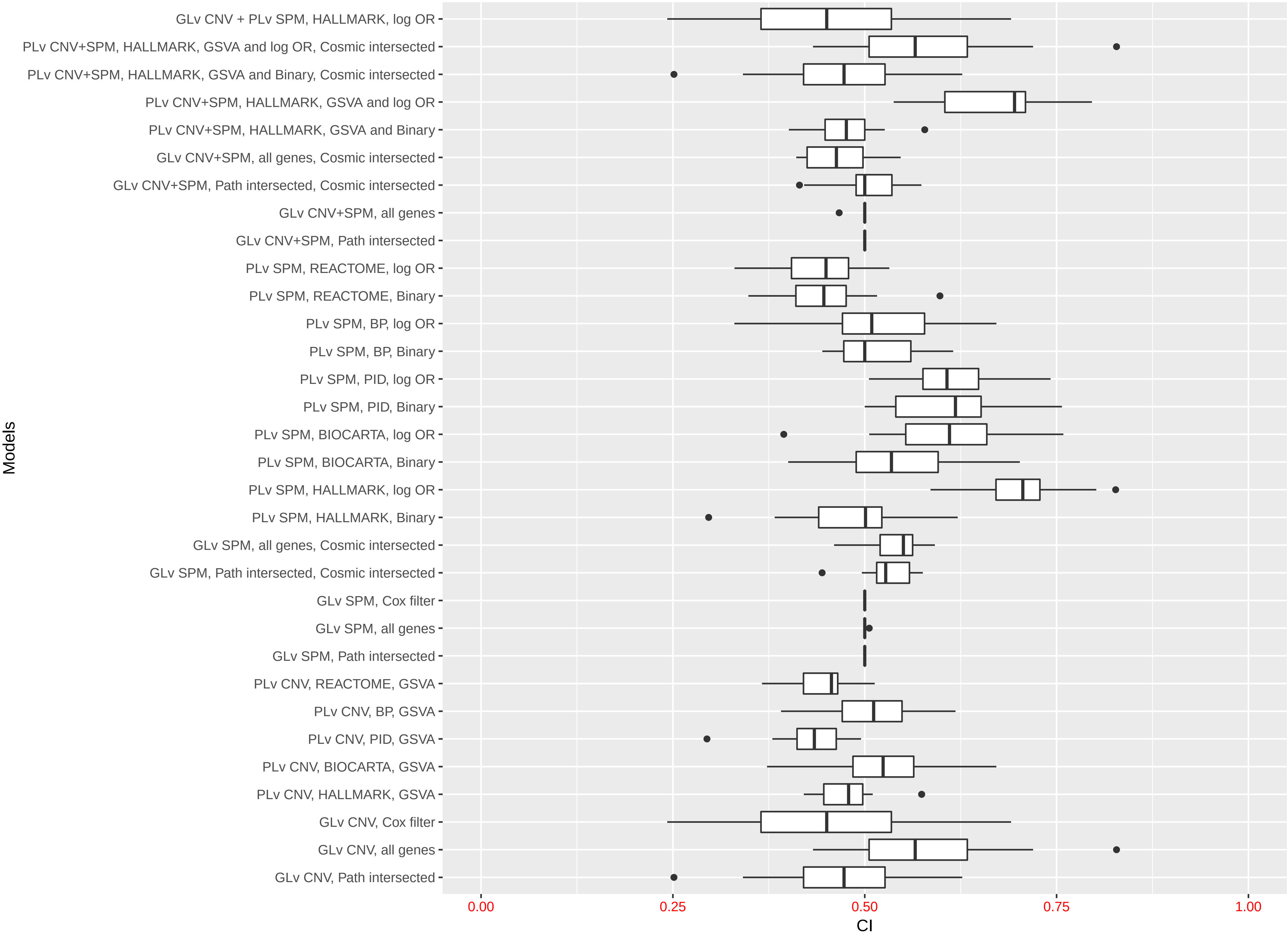

Figure S28 THYM cohort

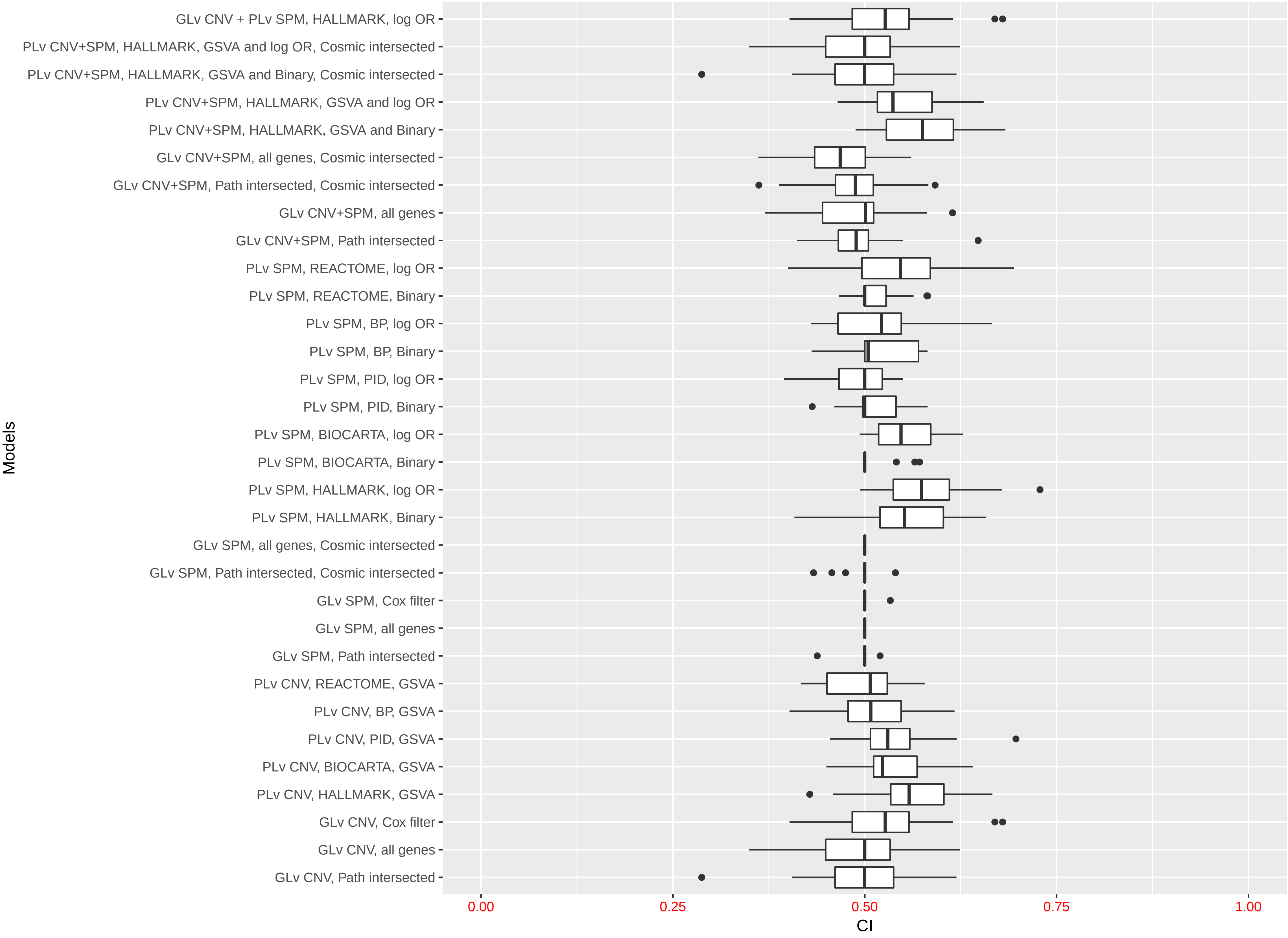

Figure S29 UCEC cohort

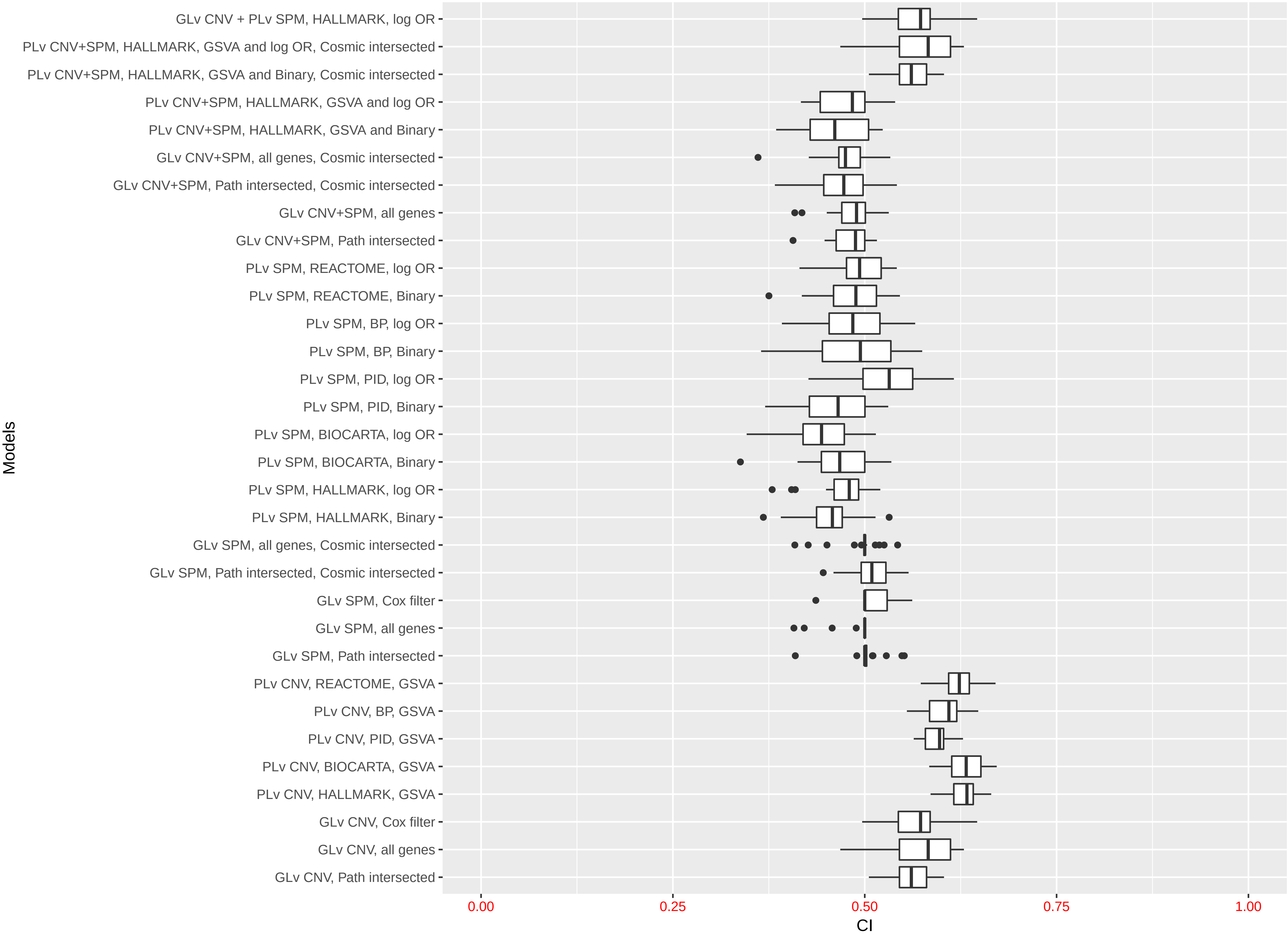

Figure S30 UVM cohort

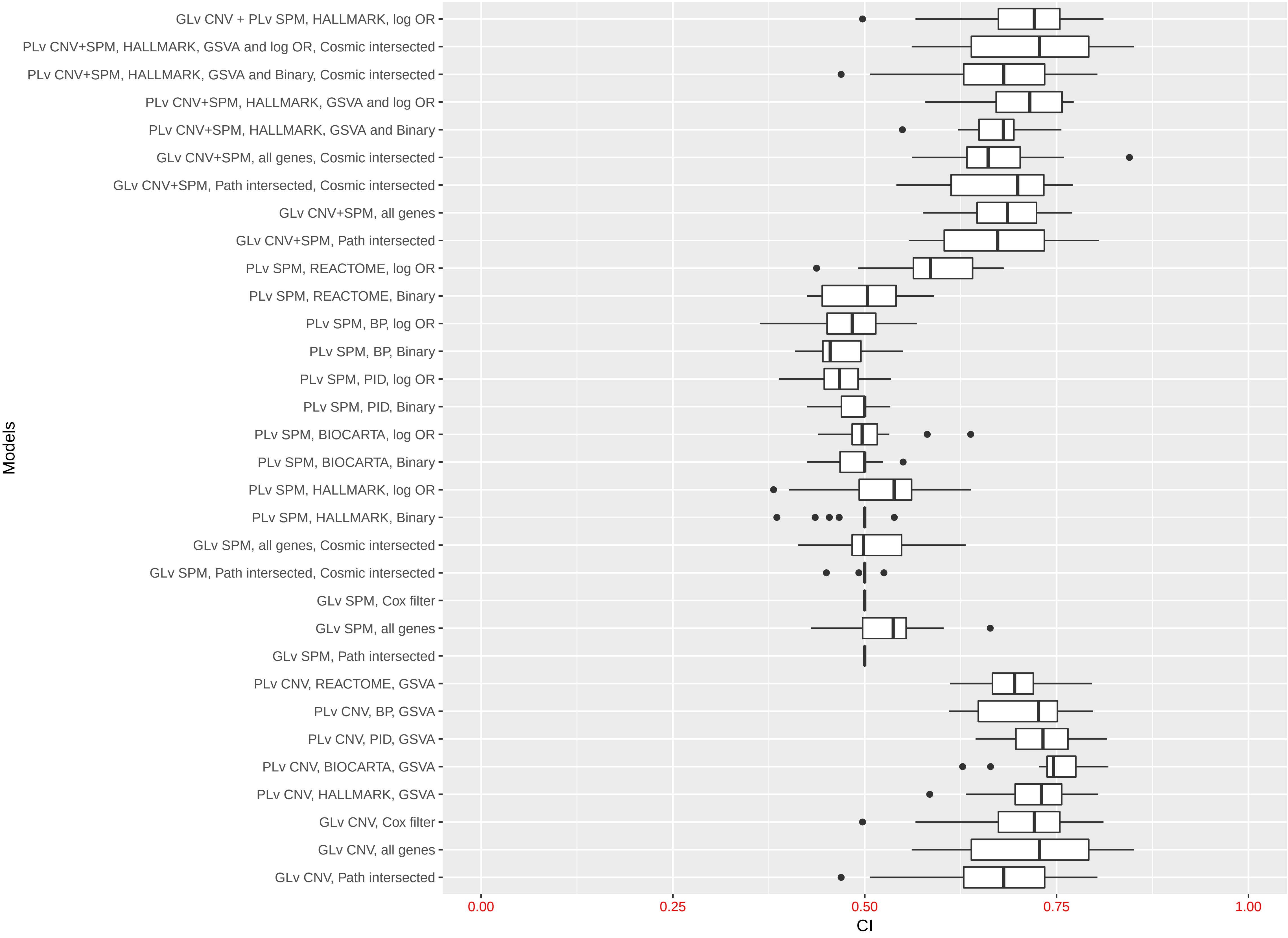

Results of simulation study for Lasso

To show how Lasso works when there are duplicated variables (perfect collinearity) in the data, we designed a simple simulation study.

1000 observations of 50 variables were randomly drawn from standard normal distribution. 5 variables were true predictors of outcomes with the association that outcomes were the sums of values of these 5 variables and random noises:

$$y_j = \sum_{i=1}^{i=5} P_i + \varepsilon_j$$

i (from 1 to 50) denotes the variables and P_1 ... P_5 are the 5 true predictors. j (from 1 to 1000) denotes the observations. ε is the random noise drawn from standard normal distribution.

To simulate the perfect collinearity situation, we duplicated the 5 true predictors P_1 ... P_5 as P_{1_rep} ... P_{5_rep} and added them into the data. With this simulation data, we fitted Lasso with the shrinkage parameter lambda chosen by cross validation.

Figure S31 below shows the solution path of fitted model. Each curve corresponds to a variable. It shows the path of coefficients against the varied lambda. The two vertical cutoff lines indicate lambda value that gives minimum mean cross-validated error (left panel) and that gives the most regularized model such that error is within one standard error of the minimum (right panel). As we can see from Figure S31, Lasso was not guaranteed to discard duplicated variables no matter which lambda we chose.

The R Markdown file of this simulation study is also attached after Figure S31.

Figure S31 Solution Path

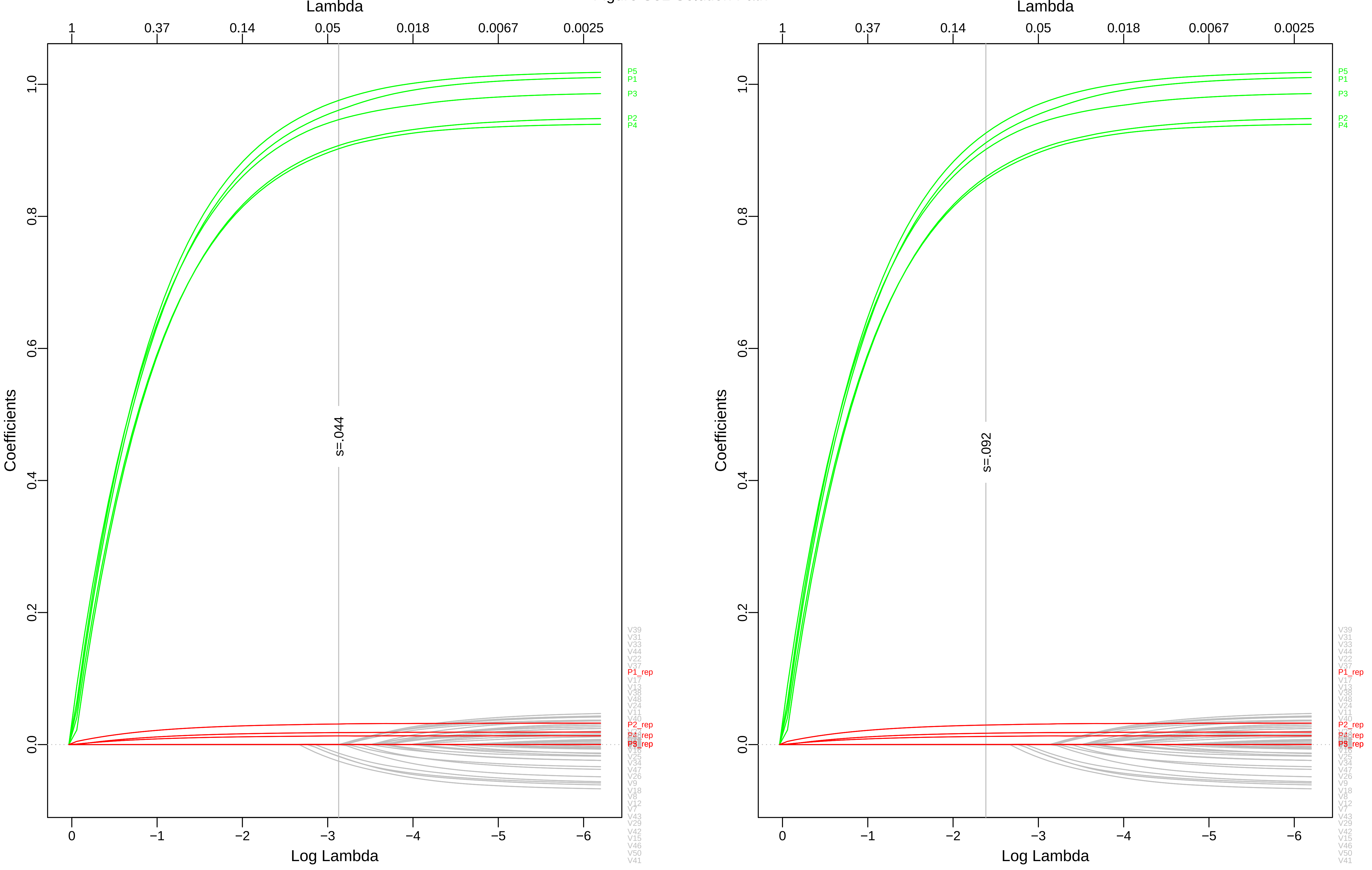

Lasso simulation Rmarkdown

This is to show how penalized regression work when there are duplicated variables.

```
library("glmnet")
```

```
## Loading required package: Matrix

## Loaded glmnet 3.0-2
```

```
library(plotmo)
```

```
## Loading required package: Formula

## Loading required package: plotrix

## Loading required package: TeachingDemos
```

```
set.seed(99999)
n <- 1000  # Number of observations.
p <- 50  # Number of variables.
real_p <- 5  # Number of true predictors.

# Generate data with strandard normal distribution.
x <- matrix(rnorm(n*p), nrow=n, ncol=p)

# Generate outcomes based on true predictors.
y <- apply(x[,1:real_p], 1, sum) +rnorm(n)

# Assign variable names.
colnames(x)<-c(paste("V",seq(1,p),sep=""))
colnames(x)[1:real_p]<-c(paste("P",seq(1,real_p),sep=""))

# Duplicate the predictors
x<-cbind(x,x[,1:real_p])
colnames(x)[(p+1):(p+real_p)]<-c(paste("P",seq(1,real_p),"_rep",sep=""))
```

```
# Fit Lasso model.
fit.lasso <- glmnet(x, y, family="gaussian", alpha=1)
```

```
# 10-fold Cross validation for lambda.
cv.fit.lasso<-cv.glmnet(x, y, alpha=1,family="gaussian")
```

```
# Get ordered list of variables.
allnames<-names(coef(fit.lasso)[,ncol(coef(fit.lasso))][order(coef(fit.lasso)[,ncol(coef(fit.lasso))],d[illegible]

# Remove intercept.
allnames<-setdiff(allnames,allnames[grep("Intercept",allnames)])

# Assign colors
cols<-rep("gray",length(allnames))
cols[allnames %in% c(paste("P",seq(1,real_p),sep=""))]<-"green"
cols[allnames %in% c(paste("P",seq(1,real_p),"_rep",sep=""))]<-"red"
```

```r
# Plot solution path.
par(mfrow=c(1,2))
plot_glmnet(fit.lasso,label=TRUE,s=cv.fit.lasso$lambda.min,col=cols)
```

```
## Warning in TeachingDemos::spread.labs(beta[iname, ncol(beta)], mindiff = 1.2 * :
## Maximum iterations reached
```

```r
plot_glmnet(fit.lasso,label=TRUE,s=cv.fit.lasso$lambda.1se,col=cols)
```

```
## Warning in TeachingDemos::spread.labs(beta[iname, ncol(beta)], mindiff = 1.2 * :
## Maximum iterations reached
```

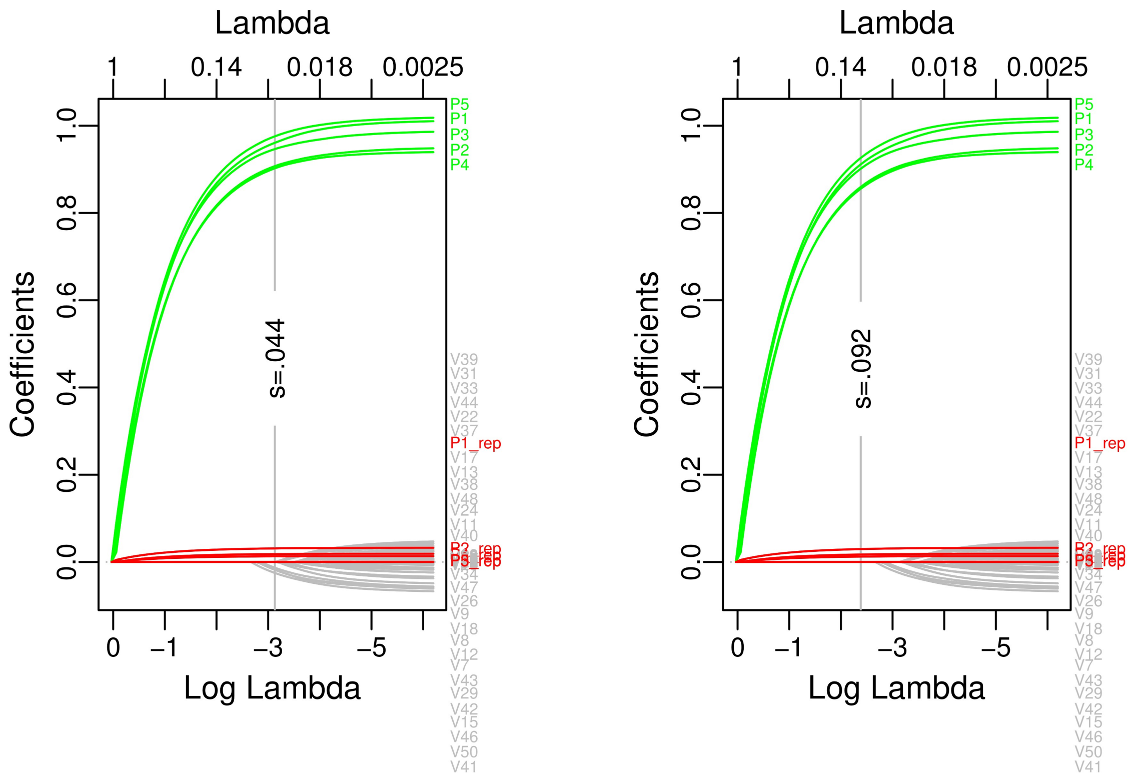

Supplement: Supplementary file 1 — Additional file 1: This file contains information on the TCGA cohorts and pathway collections used in this study and supplemental results for both the simulation and real data analyses. [file 12859_2020_3791_MOESM1_ESM.pdf]
